# Deciphering Pigmented Rice Varieties as Sustainable and Unexplored Valuable Sources of Bioactive Components With Health‐Related Properties and Technological Applications—A Systematic Review

**DOI:** 10.1111/1541-4337.70355

**Published:** 2025-12-08

**Authors:** Adolfo Pinheiro de Oliveira, Thatyane Mariano Rodrigues de Albuquerque, Evandro Leite de Souza

**Affiliations:** ^1^ Department of Nutrition, Health Sciences Center Federal University of Paraíba João Pessoa Paraíba Brazil

**Keywords:** bioactive compounds, functional properties, health, pigmented rice, technological properties

## Abstract

The interest in pigmented grains has grown substantially in recent years. Pigmented rice is crucial for diversifying agricultural production and promoting healthier, more balanced diets. This systematic review updates and discusses literature published between 2019 and 2024 on the bioactive compounds in pigmented rice, their potential health benefits, and technological applications, based on a final sample of 69 articles. Data indicate that pigmented rice varieties have valuable nutritional composition, and their by‐products have several constituents that promote health and prevent chronic diseases. The health‐related functions of flavonoids, anthocyanins, proanthocyanidins, phenolic acids, resistant starch, dietary fiber, essential fatty acids, and proteins typically found in pigmented rice and its by‐products have been confirmed through in vitro and in vivo studies, showcasing anti‐inflammatory, anticancer, antioxidant, antidiabetic, and antimicrobial activities. Several varieties of pigmented rice, including black, red, and purple, differ in their nutrient and bioactive compound content and, consequently, in their potential consumer benefits. The presence of these bioactive compounds also improved the technological properties of pigmented rice, such as stability, viscosity, and texture, and favored the development of biodegradable packaging films. These insights support considering pigmented rice varieties as healthy and sustainable food choices for domestic meal preparation and for use by the food industry as functional ingredients to formulate novel, added‐value functional food products.

## 1 Introduction

Rice (*Oryza sativa* L.) is a staple crop and one of the primary food sources worldwide. It forms the basis of the diet for nearly half of the global population (FAOSTAT 2022) and accounts for approximately 25% of global grain production (Beaulieu et al. 2023). On the basis of pigment characteristics, pigmented rice is categorized into black, red, and purple varieties of the *Oryza* genus (Bhat et al. 2020). Although the origin of various pigmented rice varieties remains unidentified, many come from Asian regions, such as India, China, and Thailand. These rice types have been cultivated for centuries and play a vital role in the cuisine and culture of these areas (Pang et al. 2018). The significance of pigmented rice varieties goes beyond their nutritional benefits, as they are crucial for diversifying agricultural production and promoting healthier and more balanced diets (Cañizares et al. 2024).

Pigmented rice varieties have gained popularity due to their high nutritional value and abundance of bioactive compounds, mainly phenolic compounds such as anthocyanins (Gong et al. 2020; Deng et al. 2013). Consumer demand for pigmented grains has grown significantly. Their nutritional value, driven by various bioactive compounds, sets them apart from nonpigmented rice. These compounds enhance the potential of these grains as functional foods that promote health and prevent chronic diseases (Suvi et al. 2020).

Pigmented rice could be a valuable source of nutrients and functional components, including dietary fiber, resistant starch (RS), minerals, vitamins, functional lipids, phytochemicals, and γ‐aminobutyric acid (Sharma et al. 2020). This wealth of nutrients and bioactive compounds makes pigmented rice a key food for maintaining a balanced and healthy diet (Rebeira et al. 2022), linked to health‐beneficial properties, such as anti‐inflammatory effects (Qiu et al. 2021), anticancer benefits (Mbanjo et al. 2020), and antioxidant and antidiabetic properties (Boue et al. 2016; Mbanjo et al. 2020).

Rice processing involves several steps, including hulling, milling, and polishing, during which approximately 30% of the grain is converted into by‐products: 20% hulls and 10% bran, resulting in roughly 70% of refined rice available for human consumption (Peanparkdee and Iwamoto 2019). Furthermore, processing directly influences the nutritional value of the grain, leading to notable losses and an increase in by‐product generation (Chen et al. 2024). These by‐products, often viewed as waste, negatively impact the environment (Feng et al. 2022), although they are generally repurposed as animal feed. In that regard, the application of bioactive compounds of pigmented rice grain, bran, and extracts has been an alternative to improve technological properties for developing functional foods and biodegradable packaging films (Ge et al. 2020).

The increasing demand for more nutritious and sustainable food products highlights the necessity of feeding a constantly growing global population while addressing concerns about health, sustainability, and environmental impact. In this context, pigmented rice varieties present a promising solution as they can be utilized fully, reducing waste generation. In recent years, the research volume on pigmented rice and its by‐products has risen significantly, underscoring its potential as a functional food. This review aims to comprehensively analyze the current literature on the functionality of different pigmented rice varieties, emphasizing their bioactive components, potential health‐related biological effects, and technological applications.

## 2 Methodology

**FIGURE 1:**
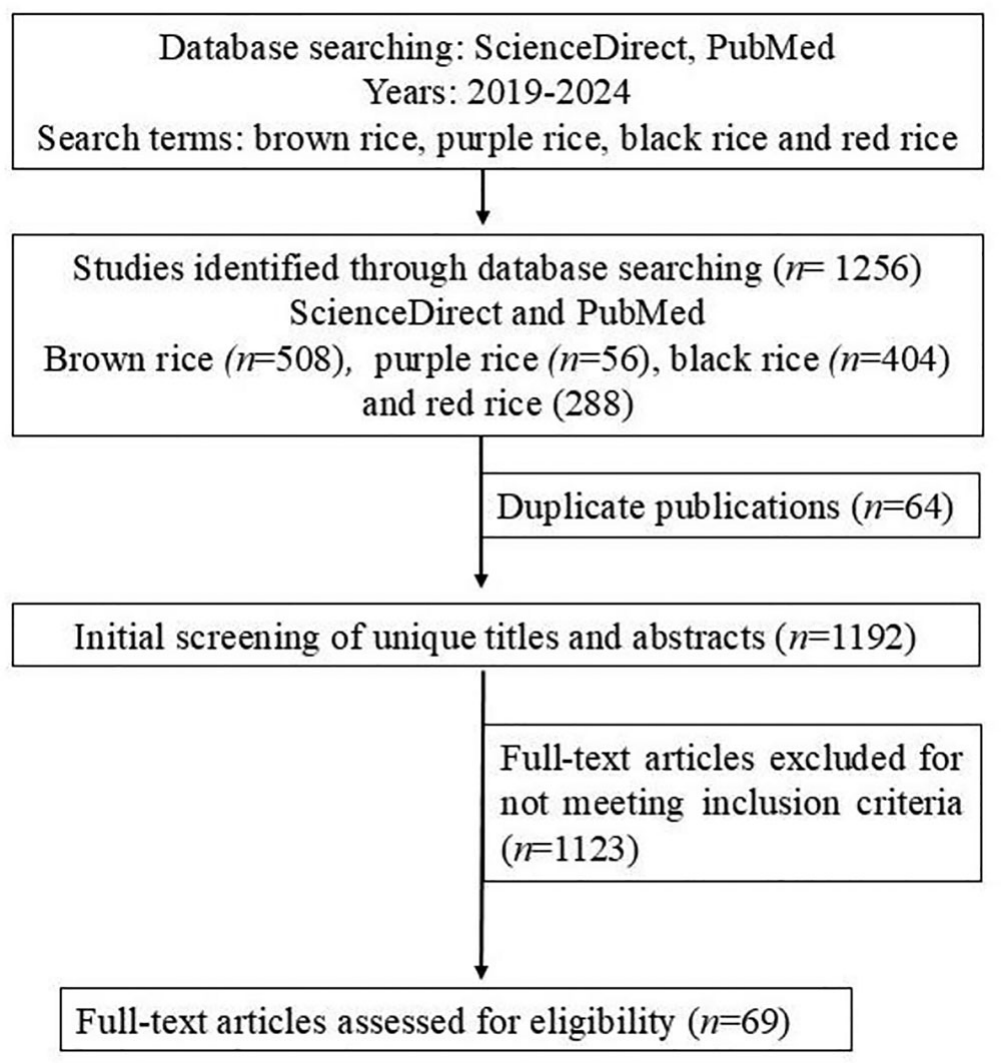
Flowchart of the literature search and selection process, showing identification, screening, and inclusion of 69 articles.

A systematic review was carried out using the Science Direct and PubMed databases, covering publications between March 2019 and November 2024. The search used the terms brown rice, purple rice, black rice, and red rice, with filters applied to titles, abstracts, and keywords. The initial search identified 1256 articles: 508 on brown rice, 56 on purple rice, 404 on black rice, and 288 on red rice. After excluding 64 duplicates, 1192 articles remained for screening, and 69 articles composed the final sample (Figure 1).

The predefined inclusion criteria considered original studies in scientific journals, available in full text, written in English, published between March 2019 and November 2024. Studies specifically addressing pigmented rice varieties (purple rice, black rice, and red rice) in whole grain, flour, and bran form and presenting data on chemical composition, functional properties, biological effects, and applications in health and nutrition were included. Duplicate articles, non‐peer‐reviewed papers, conference abstracts, theses, dissertations, and studies whose focus was not directly related to the review objective were excluded. This process yielded a final sample of 69 articles, categorized as follows: 21 studies addressed the bioactive components of pigmented rice varieties; 19 provided in vitro and in vivo evidence from animal models regarding their functional effects; 14 investigated the impact of pigmented rice varieties through human in vivo studies; and 15 explored technological aspects related to the application of these rice varieties.

Data were systematically extracted and organized into a standardized format, which included the studied pigmented rice varieties (black, red, and purple), study objectives, analyzed parameters, primary findings, and authorship. The inclusion criteria focused on studies discussing the functional properties of pigmented rice varieties and providing in vitro or in vivo evidence. Articles that exclusively addressed nonpigmented rice or were not aligned with the research objective were excluded.

## 3 Bioactive Components of Pigmented Rice Varieties

Foods contain nutrients and bioactive components, including lipids, peptides, and antioxidants essential to human nutrition. Functional foods provide health benefits beyond basic nutritional roles (Galanakis 2021). Whole vegetables exemplify this category, as they are abundant in bioactive compounds that help protect cells from oxidative damage, reducing the risk of chronic diseases (Eker et al. 2019).

**FIGURE 2:**
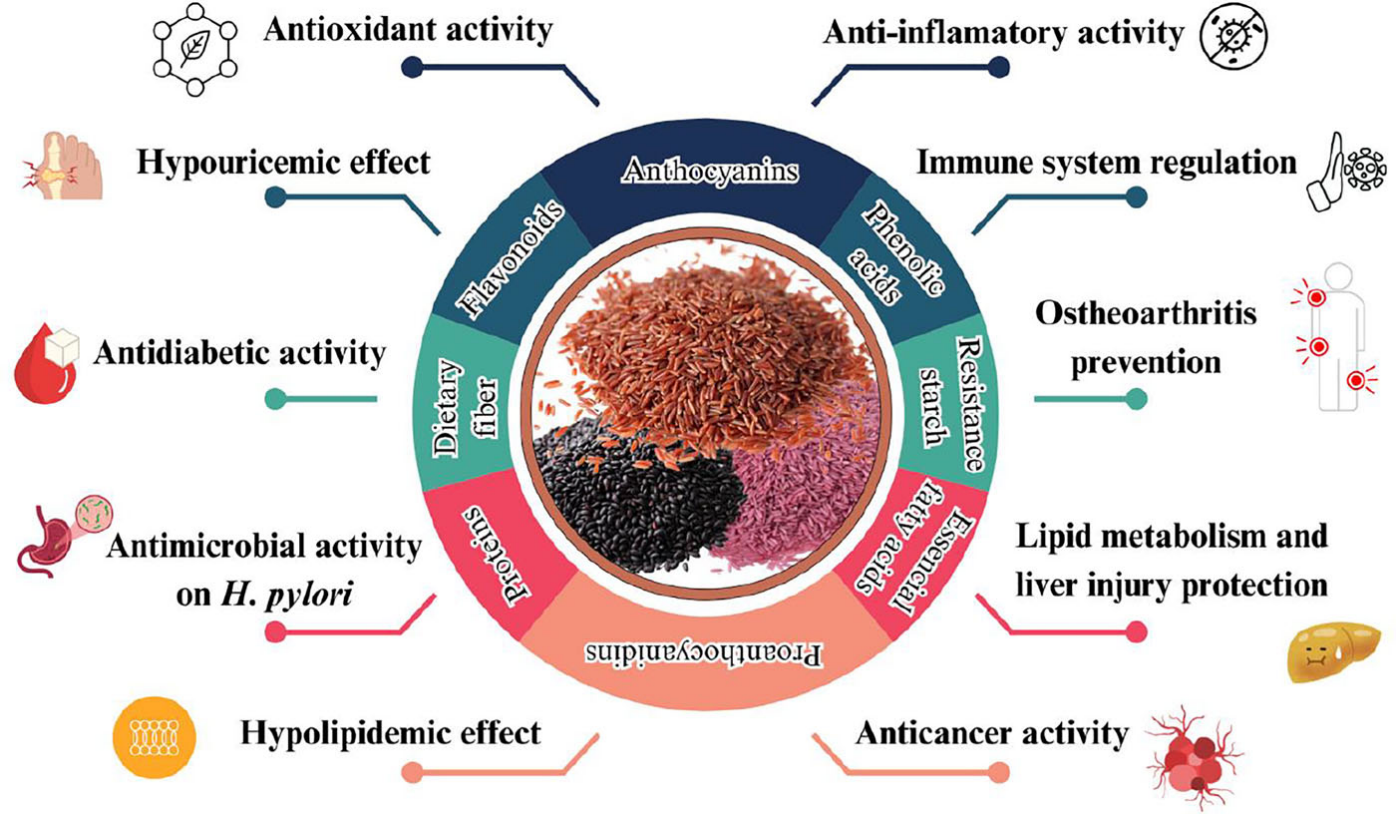
Health‐related effects of bioactive compounds in pigmented rice, including antioxidant, anti‐inflammatory, antimicrobial, antidiabetic, hypolipidemic, and anticancer activities.

Pigmented rice varieties have attracted attention due to their rich composition of bioactive compounds, which have been associated with various health benefits (Figure 2). These functional components are primarily concentrated in the outer layers of rice grains and differ by pigment type, including black, red, and purple rice. Although several studies highlight the potential of these bioactive compounds in disease prevention, it is crucial to critically examine their bioavailability, stability, and efficacy in vivo. Table 1 comprehensively summarizes studies investigating these bioactive components, emphasizing their potential as functional foods for health promotion and disease prevention.

**TABLE 1:** Bioactive compounds and biological effects of pigmented rice varieties.

Species	Objective	Main results	Geographical location	Reference
Purple rice	Identify metabolites responsible for antioxidant properties	Identification of anthocyanins, flavonoids, oligomeric proanthocyanidins, phenolic acids, tocopherols, and tannins as major antioxidants	Jiangsu, China	Xiong, Sun, et al. (2022)
Purple rice	Analyze the impact of polishing on nutrients and minerals	Significant loss of bioactive compounds (anthocyanins, flavonoids) and essential minerals (iron, zinc, and magnesium) in polished rice	Jiangsu, China	Xiong, Wang, et al. (2022)
Purple rice	Explore changes in nutrients and mineral elements and the relationship with metabolites in polished rice	Polishing resulted in an overall reduction in the nutritional value of purple rice, especially in antioxidant compounds and micronutrients	Jiangsu, China	Xiong et al. (2023)
Purple rice	Evaluate phenolic compounds and their bioaccessibility after simulated digestion	Phenolic‐rich composition but with reduced bioaccessibility and antioxidant activity after simulated gastrointestinal digestion	Mackay, Australia	Nignpense et al. (2022)
Purple rice	Investigate the effects of grinding on flavonoid content and antioxidant capacity	Reduction in the content of flavonoids, oligomeric proanthocyanidins, and total phenols, decreasing the antioxidant capacity	Guangling, China	Zhu et al. (2022)
Purple rice	Analyze digestibility and prebiotic potential after fermentation	Germinated rice showed greater digestibility and prebiotic potential compared to hydrated rice, with preservation of bioactive compounds	Songkhla, Thailand	Owolabi et al. (2020)
Purple and red rice	Evaluate the amylolysis and digestibility of starches	Purple rice has higher resistant starch and anthocyanins but lower digestibility than red rice	Surin and Phatthalung, Thailand	Ratseewo et al. (2019)
Black rice	Analyze phenolic profiles and bioactivity in bran fractions	Less processed fractions presented higher phenolic content, antioxidant, anti‐inflammatory, and antiproliferative activities	Shandong, China	Zhang, Ma et al. (2022)
Black rice	Assess the impact of processing on antioxidant conservation	Puffing processing retained more bioactive compounds and greater antioxidant activity compared to cooking	Cuttack, India	Bagchi et al. (2021)
Black rice	Examine phenolic compounds and biological activities after processing	Increased ferulic acid and starch digestibility after processing	Liaoning, China	Aalim et al. (2021)
Black rice	Characterize the bran and hypoglycemic activity	Bran rich in soluble fiber and phenolics inhibits the action of α‐amylase	Guangzhou, China	Hu et al. (2022)
Black and red rice	Investigate flavonoid compounds and antioxidant capacity	Black rice presented higher levels of anthocyanins and flavonoids, resulting in superior antioxidant capacity compared to red rice	Sichuan, China	Chen, Yang, et al. (2022)
Black and red rice	Evaluate functional properties of brown rice flour	Both rich in phenolic acids and flavonoids; black rice with higher anthocyanin content and antioxidant capacity	Ubon Ratchathani and Amnat Charoen, Thailand	Waewkum and Singthong (2021)
Black and red rice	Identify antioxidant and mineral contents	Red rice with higher content of total phenolics, flavonoids, zinc and iron; black rice with more anthocyanins and proteins	India	Singh et al. (2022)
Black and red rice	Evaluate antioxidant properties of pigmented rice cultivars	Black rice had higher concentrations of anthocyanins and flavonoids, with the highest total antioxidant activity. Red rice, although with less anthocyanins, showed high levels of flavonoids and significant antioxidant activity	Tamil Nadu, India	Meera et al. (2019)
Black and red rice	Investigate and compare the composition and levels of primary and secondary metabolites	Black rice showed higher mean concentrations of anthocyanins, flavonoids, and phenolic acids compared to red rice, reflecting greater antioxidant activity	Beijing, China	Zhang, Cui, et al. (2023)
Black and red rice	Evaluate the content of bioactive compounds in raw and cooked pigmented rice	Cooking resulted in a significant decrease in the levels of carotenoids, anthocyanins, and free phenolic compounds, whereas insoluble phenolic compounds had a smaller reduction	Italy and France	Melini et al. (2019)
Black and red rice	Identify differences between varieties and correlation between phenolics, flavonoids, proanthocyanidins, and antioxidant activity	Black rice showed a higher content of phenolic and flavonoid compounds, in addition to superior antioxidant activity compared to red rice	Huaian and Jingzhou, China	Yu et al. (2020)
Black, red and brown rice	Optimize fermentation conditions for sustainable food production and consumption of fermented rice water using pigmented rice, followed by analyzing the nutritional, physicochemical, and phytochemical properties	Black fermented rice water showed the highest phenolic and antioxidant activity, maximum bioaccessibility of polyphenols, microbiologically safe, stable during a storage period of 30 days at 4°C, in addition to the highest lactic acid bacteria count	Tamil Nadu, India	Mishra et al. (2024)
Red rice	Investigate the effects of dietary fiber and polyphenols derived from red rise grown in two geological regions on the gut microbiome and metabolome	Red rice from Thailand showed higher phenolics and anthocyanin content. Both red rice favored the growth of beneficial bacteria and showed diverging degree of short‐chain fatty acid production	Mappillai Samba, India and Sangyod, Thailand	Chakkaravarthi et al. (2025)
Red rice	Evaluate the effects of heat and moisture treatment on starch properties	Increased digestibility and insulin response, both in vivo and in vitro	Ben Tre, Vietnam	Van Hung et al. (2020)
Red rice	Compare phytochemical properties of raw and cooked samples	Even after thermal processing, red rice remained superior in bioactive compounds and antioxidant capacity	Tamil Nadu, India	Nayeem et al. (2021)
Red rice	Evaluate the effect of saline stress on pigment accumulation	Moderate saline stress increased the content of bioactive pigments, enhancing antioxidant properties	Anqing, China	Yan et al. (2023)

**TABLE 2:** Evidence of technological applications of pigmented rice.

Pigmented rice	Objective	Parameters	Main results	References
Purple red rice bran	Investigate the effects of anthocyanin extracts on rice starch properties	Antioxidant activity, resistant starch content, enzyme activity, and molecular interactions	Purple red rice bran anthocyanin extract improves antioxidant properties, increases resistant starch, reduces starch digestibility by forming intrahelical V‐type complexes and altering enzyme structures, particularly via interactions with aromatic amino acids (Phe and Tyr)	Zhang, Wang, et al. (2023)
Purple red rice bran (PRRBA)	Investigate the effect of PRRBA on the physicochemical, rheological, and gel properties of rice starch	Pasting parameters, rheological properties, gel strength, water‐holding capacity, and particle size distribution	PRRBA decreases peak viscosity (PV), final viscosity (FV), and activation energy of rice starch; enhances gel strength, water‐holding capacity, and particle size distribution. PRRBA interacts with starch through non‐covalent bonds, improving starch structure and retrogradation	Zhang, Zhu, et al. (2023)
Purple rice extract (PRE) and black rice extract (BRE)	Develop antioxidant and pH‐sensitive films by incorporating PRE or BRE into chitosan (CS) matrices	Extract content: 1%, 3%, and 5% w/w structural, physical, and functional properties assessment	1% extract content: compatible with film matrix; higher content disrupts homogeneity. Increased thickness, light barrier, and antioxidant activity with extract addition. pH‐sensitive due to anthocyanins; more pronounced color change in pork spoilage monitoring	Yong et al. (2019)
Purple rice bran	Microencapsulate anthocyanin extract using modified glutinous rice starch via spray drying	Starch concentration (6.01%), inlet air temperature (168.78°C), atomizer pressure (4.96 MPa), water activity, solubility, particle size, and rheology	Improved anthocyanin stability at 4°C (90 days). Microcapsules had smooth surfaces and altered rice dough rheology by reducing pseudoplasticity (5%–20% addition)	Das et al. (2019)
Purple and red rice	To investigate the technological properties of pigmented rice	Cooking time, amylose content, water uptake ratio, hardness, gelatinization temperature, and final viscosity	– Purple rice: longest cooking time, high hardness, color loss due to anthocyanin degradation – Red rice: highest gelatinization temperature and final viscosity, suitable for thickening applications	Devi and Badwaik (2022)
Black rice extract (BRE)	Evaluate the incorporation of black rice extract (BRE) and other anthocyanin‐rich extracts into soybean isolate protein/chitosan (SPI/CS) films and assess their performance as freshness indicators	Structural and physical properties, water barrier properties, stability of membrane structure, and macromolecular interactions	BRE improved the water barrier and antioxidant properties of SPI/CS films. BRE contributed to improving the functional properties of bio‐based indicator films, although its color sensitivity was lower than that of other extracts	Xiao et al. (2023)
Black rice	To evaluate the effect of ultrasound‐assisted immersion during parboiling on head rice yield (HRY), nutritional profile, and texture of black rice	Ultrasound power: 540; immersion time: 45 min; temperature: 57°C	Ultrasound‐assisted parboiling of black rice increased head rice yield by 20% and improved cooked rice texture	Wu et al. (2023)
Black rice	Evaluate the impact of pretreatments on rice quality	Texture, water absorption, and cooking time	Enzymatic hydrolysis improved texture, reduced hardness/adhesion ratio, increased water absorption, and reduced cooking time	Xiong et al. (2023)
Black rice	To assess the effect of BRWE on ground beef quality	– Color (redness) – Lipid oxidation	– BRWE improved beef color (redness) – Reduced lipid oxidation, extending shelf life and preventing rancidity	Prommachart et al. (2020)
Black rice	Develop a pH‐sensitive, antioxidant film for food freshness monitoring	FTIR, XRD, SEM, AFM, mechanical properties, UV–Vis barrier, antioxidant activity, pH sensitivity, and color change	pH‐sensitive color change for spoilage detection; high antioxidant and UV barrier properties; reduced tensile strength; improved oxygen and moisture barrier	Ge et al. (2020)
Black rice	Development of a biodegradable intelligent packaging film incorporating black rice anthocyanins for real‐time monitoring of meat freshness	Mechanical properties, colorimetric response, and moisture resistance	Increased mechanical resistance of the pectin–chitosan composite film. Colorimetric change from red to blue with meat spoilage, indicating food freshness. Limited moisture resistance and anthocyanin stability affected by light, temperature, and pH variations	Zeng et al. (2022)
Black rice	Develop intelligent films for food packaging	pH sensitivity, UV‐barrier, antioxidant, mechanical, and barrier properties	Films showed pH‐sensitive color changes, excellent UV‐barrier, and antioxidant properties but reduced mechanical and barrier strength. COB‐3 films (3% BACNs) successfully monitored spoilage of fish and shrimp	Wu et al. (2019)
Black rice	Develop an antibacterial, intelligent packaging film	Mechanical properties, antibacterial, antioxidant, UV‐barrier, and pH sensitivity	Film showed good mechanical properties, antibacterial and antioxidant effects, and pH‐sensitive color change for freshness monitoring. Enhanced pork preservation, reduced spoilage bacteria, delayed odor	Hao et al. (2023)
Purple rice extract (PRE) and black rice extract (BRE)	Develop antioxidant and pH‐sensitive chitosan‐based packaging films	Total phenol, anthocyanin content, structural and physical properties, antioxidant activity, pH sensitivity	BRE had higher phenols and anthocyanins than PRE. 1 wt% extract improved film properties; higher levels reduced homogeneity. Films were pH‐sensitive and suitable for spoilage monitoring	Yong et al. (2019)
Black rice	To study protein distribution and its impact on starch properties	Protein distribution and pasting properties	Protein concentrated in outer layers, affects starch viscosity and pasting temperature and protein extraction weakens starch stability	Chen, Lu, et al. (2022)

Abbreviations: BACN, black rice bran anthocyanins; BRWE, black rice water extract; UV, ultraviolet.

Pigmented rice varieties offer high‐quality proteins, fiber, and a range of vitamins and are recognized for their functional properties. They contain a high concentration of antioxidant compounds that inhibit reactive free radicals and safeguard cells against oxidative damage (Bhat et al. 2020). The primary antioxidants include anthocyanins, such as polymeric proanthocyanidins, and anthocyanidins, such as cyanidin and malvidin (Zhuang et al. 2019). Additionally, phenolic compounds, such as syringaldehyde, anisole, *p*‐coumaric acid, protocatechuic acid, vanillin, ferulic acid, and sinapinic acid, are present in pigmented rice varieties, contributing to their antioxidative effects (Mbanjo et al. 2020). Variations in the concentration and efficacy of these compounds due to differences in rice processing, storage conditions, and cooking methods must be considered when assessing their actual functional impact.

Phenolic compounds play a crucial role in preventing chronic diseases, like cardiovascular disease (CVD) and Type 2 diabetes, primarily due to their antioxidant properties, and their actual physiological effects depend on their metabolic fate in the human body. However, their bioavailability is influenced by several factors, including the rice variety, environmental conditions, and postharvest treatments (Meera et al. 2019). It is necessary to clarify how different processing techniques affect the retention and absorption of these bioactive compounds.

Anthocyanins, flavonoid compounds responsible for black, red, and purple rice colors, are primarily found in the bran of pigmented grains. The main aglycones identified include cyanidin, peonidin, and delphinidin, which are predominantly located in the pericarp layer and aleurone in black and purple rice. Conversely, the distinctive color of red rice is mainly attributed to proanthocyanidins, which belong to a different class of flavonoids (Bhat et al. 2020). The stability of anthocyanins under different food processing conditions remains challenging, as they are highly susceptible to degradation due to pH changes, temperature fluctuations, and enzymatic activity. Therefore, although the health benefits of these compounds are promising, the extent to which they retain their functional properties during food preparation warrants further investigation.

Anthocyanins are known to neutralize free radicals, reduce oxidative stress, and prevent cellular damage. Scientific evidence supports their protective effects against CVDs, potentially by improving lipid profile and mitigating the inflammatory process (Mbanjo et al. 2020; Bhat et al. 2020). Additionally, these compounds may modulate inflammatory pathways, showing promise in managing chronic inflammatory diseases, such as arthritis and metabolic disorders. However, the mechanisms underlying these effects are not yet fully understood, and discrepancies in study outcomes suggest that genetic and environmental factors may influence their bioactivity. For instance, although some research indicates that black rice anthocyanins enhance phagocytosis and immune response, variations in study design and sample populations complicate direct comparisons (Bhat et al. 2020).

Rice varieties with darker coats generally contain higher levels of anthocyanins, flavonoids, and other antioxidant compounds, contributing to their enhanced ability to neutralize free radicals (Chen, Lu, et al. 2022). Additionally, phenolic compounds, including hydrolyzed tannins, flavonoids, and polyphenolic complexes, are abundant in pigmented rice, further amplifying their antioxidant effects and health benefits (Xiong et al. 2023). However, translating these findings into dietary recommendations requires specific clinical trials to confirm efficacy, optimal intake levels, and long‐term health effects.

Although the bioactive compounds in pigmented rice varieties present a compelling case for their inclusion in functional food strategies, a more nuanced approach is needed to evaluate their health benefits. Future research should focus on elucidating the mechanisms of action, optimizing processing techniques to retain bioactivity, and conducting long‐term human studies to determine their clinical significance. Only through a comprehensive understanding of these factors can pigmented rice varieties be effectively positioned as functional foods with tangible health benefits.

### 3.1 Purple Rice

**FIGURE 3:**
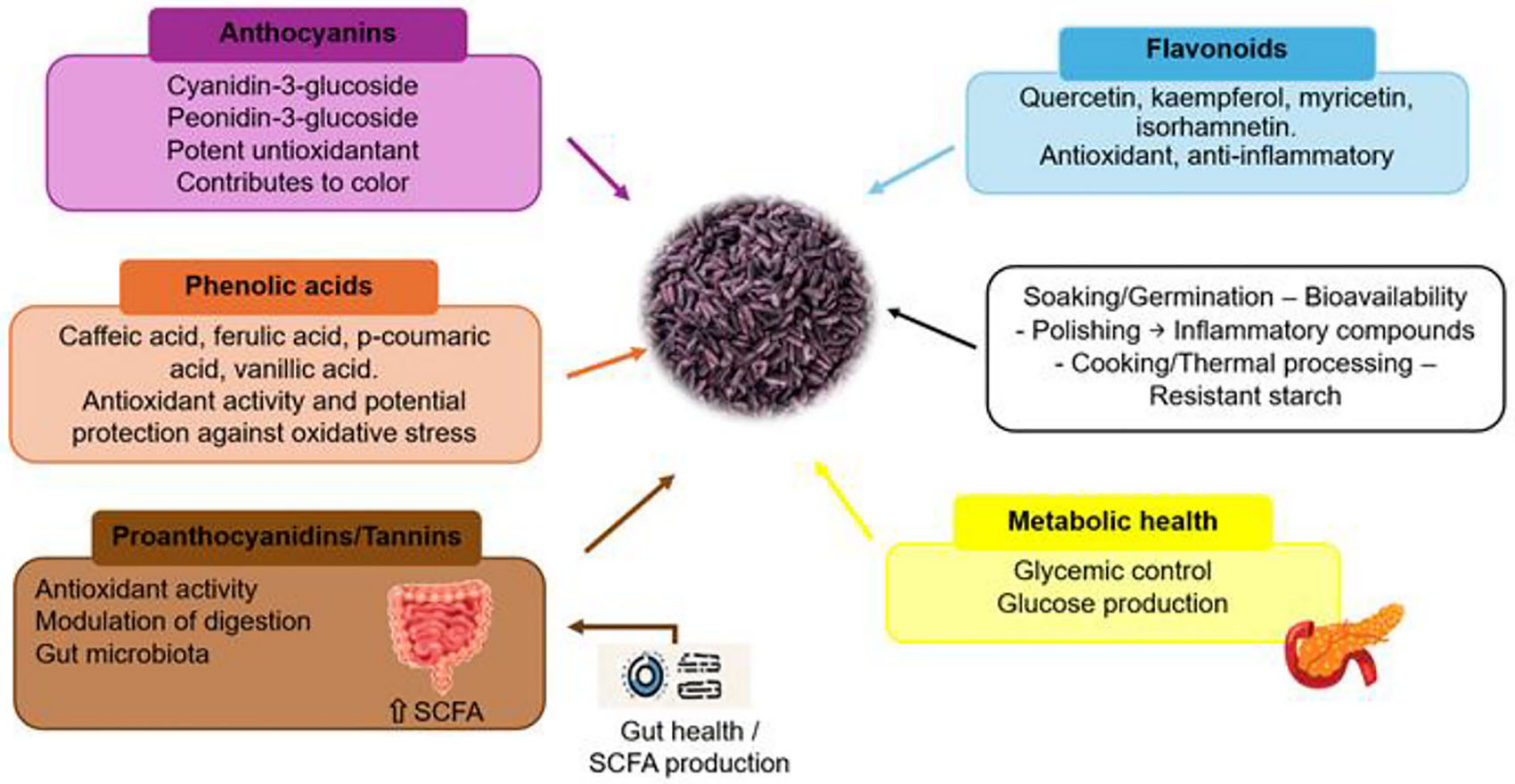
Main bioactive components of purple rice and their roles in antioxidant protection, gut health, and metabolic regulation.

During the grain‐filling phase of purple rice, several metabolites with beneficial properties are present. Phytochemicals, including anthocyanins, flavonoids, phenolic acids, and proanthocyanidins (tannins), are the main contributors to its nutritional and functional profile (Figure 3). Anthocyanins, such as cyanidin‐3‐glucoside and peonidin‐3‐glucoside, give purple rice its characteristic color and provide strong antioxidant effects. Flavonoids, including quercetin, kaempferol, myricetin, and isorhamnetin, exhibit antioxidant and anti‐inflammatory activity. At the same time, phenolic acids, such as caffeic, ferulic, *p*‐coumaric, and vanillic acids, are recognized for their potent antioxidant properties. Proanthocyanidins further enrich the phenolic composition, emphasizing the nutritional value of purple rice (Xiong et al. 2023; Nignpense et al. 2022).

The bioefficacy of these compounds depends on their stability during storage and digestion, which influences their functional contribution to human health. The antioxidant capacity of purple rice increases significantly during the grain‐filling period, reaching 65%, indicating that its bioactive compounds play an essential role in protecting against oxidative stress. However, it remains unclear whether this increase translates into tangible health benefits upon consumption, due to potential losses during digestion and processing. Additionally, although anthocyanins and flavonoids are key metabolites, their bioavailability and metabolic fate require further investigation to confirm their functional relevance in humans (Xiong et al. 2023).

Processing brown purple rice into polished rice markedly reduces the concentrations of bioactive compounds, minerals, and fibers, as the outer layers of the grain (pericarp, testa, and aleurone) are removed. Although excessive washing or overcooking may contribute to nutrient loss, polishing is the primary factor, physically eliminating nutrient‐rich fractions. Consequently, polished rice shows lower anthocyanin and flavonoid content, reduced levels of essential minerals, such as iron and zinc, and decreased antioxidant capacity compared to brown purple rice (Xiong et al. 2023; Zhu et al. 2022). While polishing improves sensory attributes and shelf life, it compromises the functional properties of rice. Strategies such as partial polishing or fortification should be considered to preserve nutritional quality while maintaining consumer acceptance.

Simulated gastrointestinal digestion enhances the bioaccessibility of phenolic compounds in purple rice, especially ferulic acid and quercetin, whose chemical structure facilitates absorption, potentially preserving their antioxidant activity. However, some phenolic compounds undergo degradation, leading to a shift in the profile of compounds that the body can effectively absorb (Nignpense et al. 2022). Although the overall antioxidant activity remains significant after digestion, reductions in quercetin and kaempferol activity indicate a potential limitation in their functional benefits. These findings underscore the need for in vivo studies to determine whether enhanced bioaccessibility translates into meaningful health outcomes.

Soaking and germinating purple rice enhances the availability of bioactive compounds, improving their bioaccessibility after digestion. Ferulic acid demonstrated a 30% increase in bioaccessibility, whereas cyanidin‐3‐glucoside decreased by 40%. Additionally, kaempferol declined by about 15% after metabolism by the intestinal microbiota, whereas quercetin was converted into antioxidant metabolites, potentially boosting its bioactivity. The production of short‐chain fatty acids increased by 25%, suggesting benefits for intestinal health. Furthermore, the antioxidant activity of the metabolized compounds remained, with a 20% increase in total antioxidant capacity (Owolabi et al. 2020). These findings indicate that pretreatment methods, such as soaking and germination, could be strategically utilized to enhance the functional potential of purple rice. However, the variability in responses depending on individual intestinal microbiota composition highlights the complexity of dietary bioactive compounds in personalized nutrition.

RS is a portion not digested in the human upper gastrointestinal tract and can reach the large intestine, where it undergoes fermentation. RS functions similarly to soluble fiber, influencing digestion and glycemic response (Rajendran and Chandran 2020). The thermal processing of rice influences its digestibility, particularly in terms of RS content. High temperatures typically decrease RS contents, leading to quicker digestion and a higher glycemic index (GI) (Zhu et al. 2021). However, retrogradation and modified processing techniques may help maintain or enhance RS contents, crucial for developing functional rice products targeting glycemic control.

The RS content is influenced by starch structure, physicochemical properties, and grain texture (Zhu et al. 2021). Phytate levels in rice grains can bind to proteins and starch, reducing digestibility (Qi et al. 2019). Additionally, phenolic compounds, such as anthocyanins and proanthocyanidins, are crucial in modifying starch structure, increasing RS content while limiting digestibility (Zheng et al. 2021). These properties highlight the need for targeted breeding programs and technological innovations to enhance the RS content of pigmented rice varieties.

The RS content of riceberry purple rice flour was 27.1%. In contrast, Hom Mali (HM) rice flour, a Jasmine white rice (WR), has only 8.44%, highlighting the greater potential of riceberry flour to modulate glycemic response (Thiranusornkij et al. 2019). Rice bran helps protect against the degradation of rice particles, contributing to a delay in starch digestibility. The substitution of wheat flour with black rice flour has been shown to slow starch digestion and glucose release by inhibiting carbohydrate‐digesting enzymes, such as pancreatic α‐amylase and α‐glucosidase (An et al. 2016). These findings support the potential of pigmented rice varieties in formulating functional foods aimed at metabolic health.

It is important to emphasize that industrial processing, including polishing, milling, sprouting, cooking, baking, extrusion, and fermentation, can alter the nutritional profile of rice. Therefore, generalizations about the benefits of specific pigmented rice types should be made cautiously, considering the numerous variables affecting nutritional composition. Although whole grains and minimally processed rice varieties offer significant health benefits, consumer accessibility and acceptance must be factored into dietary recommendations. Optimizing these processes to balance nutritional quality with consumer preferences remains crucial for future research (Zhang, Ye et al. 2022).

### 3.2 Black Rice

**FIGURE 4:**
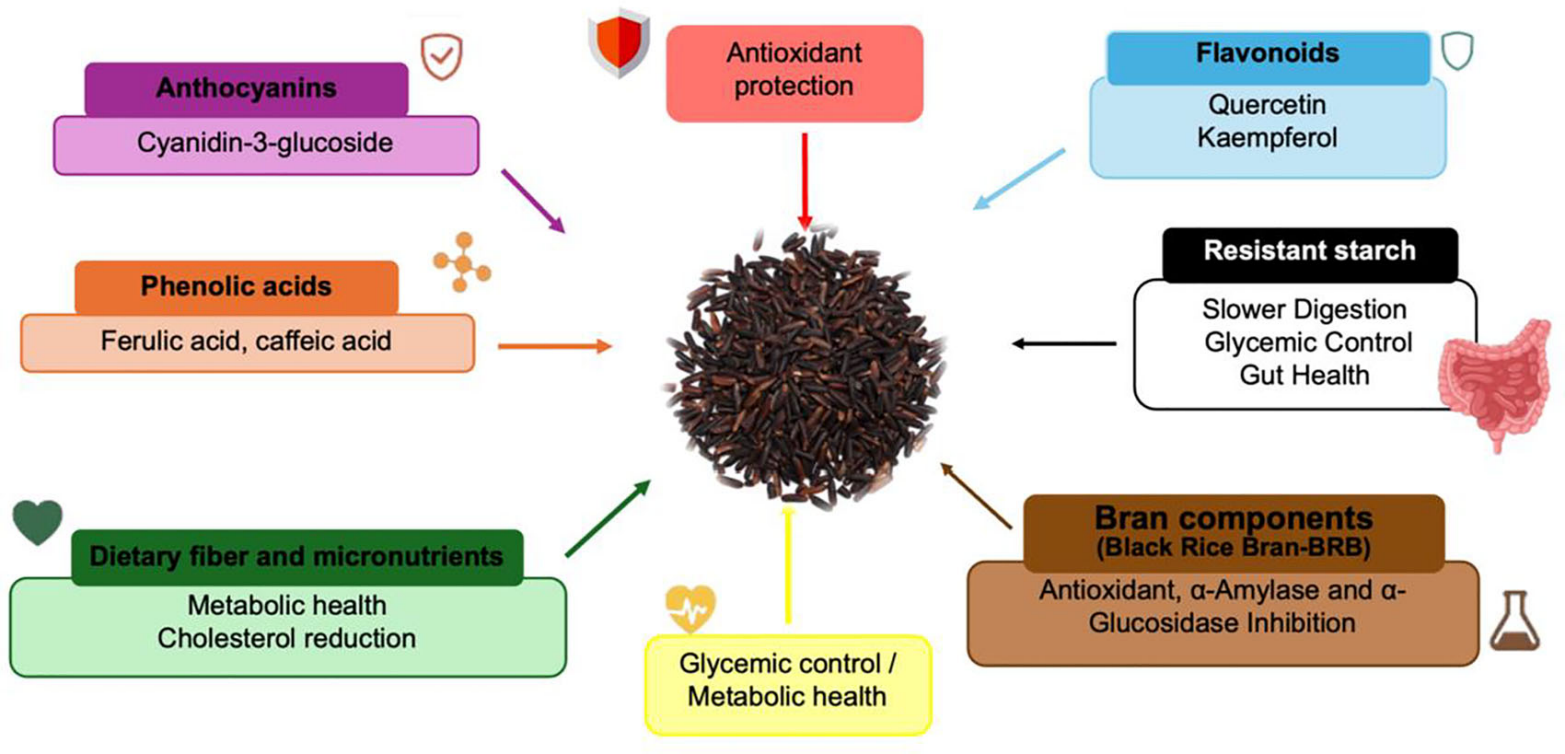
Key bioactive compounds of black rice and their contributions to antioxidant defense, glycemic control, metabolic health, and gut microbiota balance.

Black rice is a pigmented variety widely recognized for its high nutritional value and health benefits (Figure 4). Its regular consumption has been linked to a reduced risk of chronic diseases due to its high concentration of natural antioxidants, such as anthocyanins and phenolic acids. Unlike polished rice, black rice retains essential nutrients in the bran layer, including dietary fiber, vitamins, minerals, and phytochemicals, making it an exceptionally nutritious choice (Pang et al. 2018). However, the extent to which these compounds exert physiological benefits depends significantly on their bioavailability, which can be influenced by processing methods and individual metabolic responses.

The phytochemicals in black rice, particularly flavonoids, phenolic acids, and anthocyanins (Figure 4), are primarily concentrated in the aleurone layer and play a crucial role in promoting health (De Leon et al. 2023). Although these compounds have been linked to antioxidant activity and disease prevention, it is essential to critically assess the extent to which their consumption translates into measurable health outcomes in human studies.

The color of the rice pericarp reflects its anthocyanin content, with darker grains signifying higher levels of flavonoids, which correlate with antioxidant activity. This antioxidant capacity has benefits, such as preventing hypocholesterolemia, CVD, diabetes, obesity, and cancer (Massaretto et al. 2022). However, the bioefficacy of anthocyanins in black rice remains a topic of debate. Although laboratory studies highlight their antioxidant potential, their absorption, metabolism, and systemic effects in humans may be less pronounced due to rapid degradation and low bioavailability.

Nevertheless, germination can increase phenolic acid and flavonoid content by as much as 25%, enhancing overall antioxidant activity (Bagchi et al. 2021). However, they also introduce changes in sensory properties, which may affect consumer acceptance and overall dietary incorporation of black rice products. Conversely, polishing and prolonged cooking can lead to significant losses in anthocyanins and phenolic acids, with some studies indicating a reduction of up to 40% in cyanidin‐3‐glucoside in purple rice (Bagchi et al. 2021). These findings highlight a critical gap in research where there is a need to develop processing methods that optimize nutrient retention without compromising the sensory attributes of black rice. Future investigations should focus on balancing technological advancements with practical applications to maximize health benefits.

Black rice bran (BRB), often regarded as a by‐product of rice polishing, has gained attention for its potent antioxidant properties due to its high anthocyanin and fiber content (Li, Du, et al. 2023). BRB has shown promising biofunctional properties, including hypoglycemic effects through the inhibition of α‐amylase (42.58%) and α‐glucosidase (58.76%), as well as potent antioxidant activity, with inhibition rates of 78.34% (DPPH method) and 86.45% (ABTS method) (Hu et al. 2022). However, although these in vitro effects suggest potential health benefits, their translation to in vivo settings remains uncertain. Digestibility, matrix interactions, and gut microbiota metabolism need further exploration to assess the practical implications of incorporating BRB into diets.

Another relevant component of black rice is RS, which contributes to a slower release of glucose into the bloodstream, supporting glycemic control (Zheng et al. 2021). However, RS content is highly susceptible to thermal processing, which alters starch structure and reduces its functional properties (Qi et al. 2019). Research comparing different processing methods on black rice revealed substantial variations in RS content, ranging from 42.07% in boiled rice to 65.21% in fried rice (Aalim et al. 2021). Such differences highlight the potential of optimized processing techniques to preserve RS and enhance its functional benefits.

RS has gained prominence due to its functional properties, including gel‐forming capacity, swelling, viscosity, and water retention, which enhance food texture and stability (Yi et al. 2021). Its role as a dietary fiber alternative makes it valuable for formulating foods that balance nutritional benefits with consumer preference (Haldipur and Srividya 2020). Despite its potential, incorporating RS into food products must consider sensory attributes and consumer acceptance, as excessive RS levels may affect palatability and digestion kinetics. Furthermore, black glutinous rice (*O. sativa* var. glutinosa) showed a significant increase in RS content after autoclaving and freeze‐thawing, reaching 27.33% and 23.70%, respectively (Toontom and Tudpor 2022). These findings suggest that specific processing strategies can enhance RS retention in black rice, improving its nutritional and functional properties. However, it is necessary to determine whether these processing‐induced modifications influence long‐term metabolic health outcomes in diverse populations.

Although black rice offers promising health benefits due to its rich bioactive profile, further research is required to understand its bioavailability, physiological effects fully, and optimal processing methods. A more comprehensive approach, integrating clinical studies with food science innovations, will be crucial to maximizing the potential of black rice as a functional food. Future studies should also consider consumer perspectives, ensuring that technological advancements align with dietary habits and preferences to promote greater adoption of black rice in everyday diets.

### 3.3 Red Rice

**FIGURE 5:**
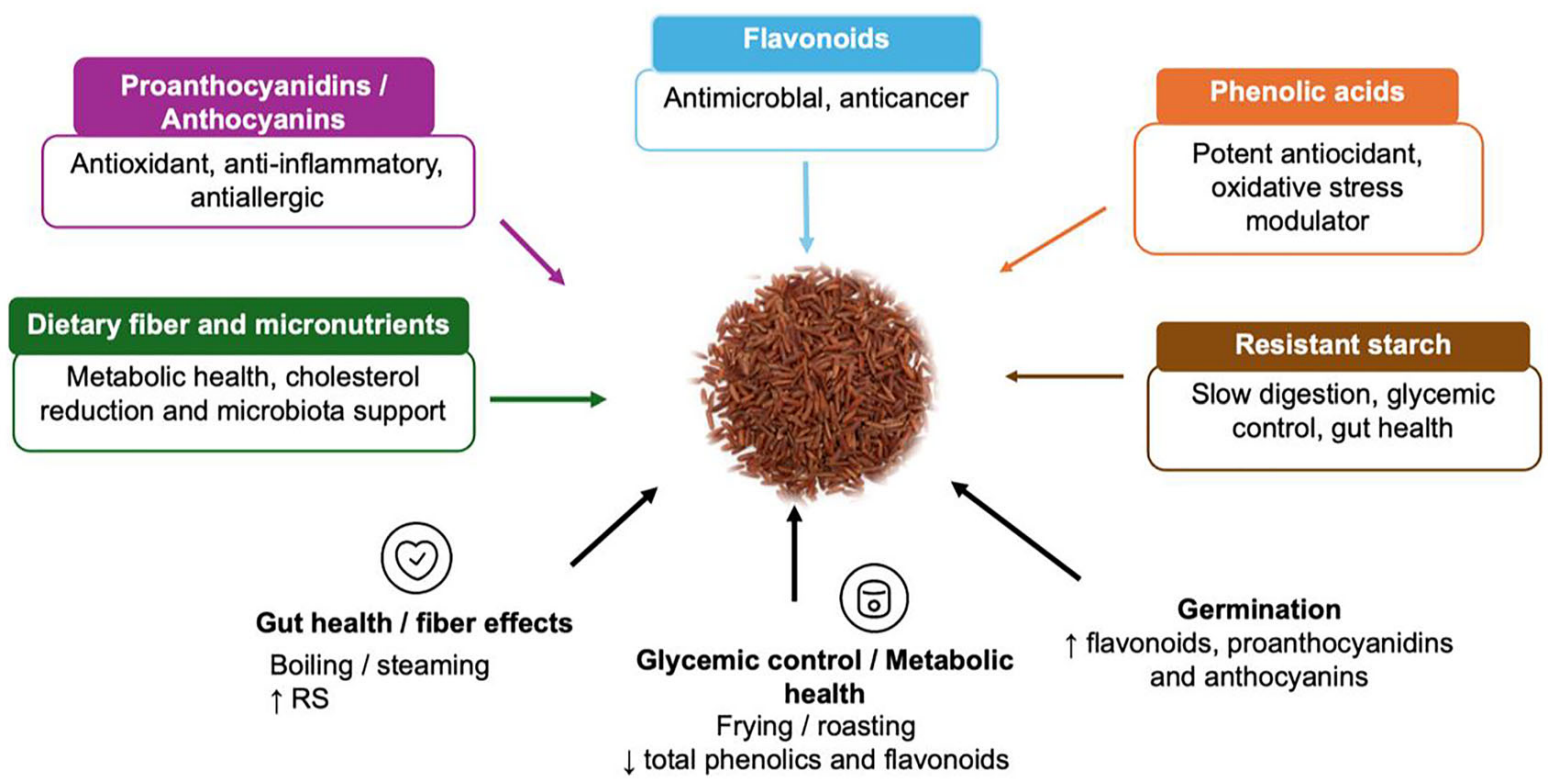
Principal bioactive components of red rice and their reported effects on antioxidant activity, glycemic control, cholesterol reduction, and gut health.

In traditional rice cultivation, red‐pigmented rice was often considered an invasive plant, which meant it was not cultivated for human consumption. However, advancements in scientific research have revealed that red rice contains high amounts of nutrients and functional components, sparking interest in its potential for human nutrition (Figure 5). These findings underscore the potential of this grain as a valuable source of nutrients and bioactive compounds, contributing to a reassessment of its role in the diet (Lang et al. 2020). Despite its nutritional benefits, red rice remains underutilized in many regions, possibly due to cultural biases and market preferences for polished WR.

Red‐pigmented rice has gained popularity due to the increasing demand for healthier dietary options and its benefits in reducing the risk of chronic diseases (Chen, Shen, et al. 2022). Compared to traditional brown rice, red rice has a higher concentration of bioactive compounds, such as flavonoids, phenolic acids, and anthocyanins, which exhibit crucial biological activities, including antioxidant, antimicrobial, anticancer, anti‐inflammatory, and antiallergic effects. Proanthocyanidins, the primary flavonoids in red rice, are responsible for the reddish hue of the pericarp and provide potent antioxidant properties (Krishnan et al. 2021). Although these compounds offer significant health benefits, their stability during processing and cooking remains a concern as it may lead to a considerable loss of phenolic compounds and flavonoids, which could limit the health‐promoting potential of red rice in real‐world dietary applications (Baptista et al. 2024).

A recent study evaluated various methods of rice preparation, including boiling, roasting, frying, and combinations of these techniques, and discovered that all of them reduced the total phenolic composition and the capacity to inhibit the enzymes α‐amylase and α‐glucosidase. Although boiling increased the RS content, which is advantageous for glycemic control, frying raised the GI (Aalim et al. 2021). These findings suggest that cooking methods play a crucial role in modulating the health effects of red rice, reinforcing the importance of selecting preparation techniques that optimize its nutritional benefits. The observed reduction in bioactive compounds during cooking calls for additional research into alternative processing methods, such as steaming or sous vide techniques, which may better preserve these beneficial components.

Rice germination is widely recognized for its positive impacts on the grain's nutritional composition and bioactive properties. Research shows notable contents of total phenolic compounds (2.1 mg GAE/g), flavonoids (0.7 mg CE/g), proanthocyanidins (0.5 mg CE/g), and anthocyanins (1.2 mg/g) in germinated red rice. These findings underscore the significance of germination as a key strategy for enhancing the nutritional and functional value of red rice (Yan et al. 2023). However, variations in germination conditions, such as time, temperature, and humidity, impact the final nutrient composition, and standardized protocols are needed to ensure consistency in functional benefits.

Comparisons between pigmented rice varieties have revealed significant differences in bioactive content, with black rice often exhibiting higher levels of total phenolic compounds and flavonoids than red rice (Chen, Yang, et al. 2022; Singh et al. 2022). Although black rice appears to have superior antioxidant potential, red rice remains a valuable alternative due to its unique composition and distinct bioactive profile. However, inconsistencies in reported phenolic and flavonoid content across different studies suggest that genetic variation, cultivation conditions, and analytical methodologies may influence the reported values (Meera et al. 2019).

Flavonoids are vital phytochemicals among secondary metabolites, present in red rice and crucial in regulating various biological functions, such as anti‐inflammatory, antimicrobial, antitumor, antioxidant, and antiallergic effects (Bhat and Riar 2017). However, black rice has higher concentrations of carotenoids, like zeaxanthin, β‐carotene, and lutein, than red rice (Melini et al. 2019). Although these differences may suggest a superior health impact of black rice, the bioaccessibility of bioactive compounds in red rice still requires further investigation.

The variation in RS content among distinct red rice varieties highlights its potential role in metabolic health. For instance, the 60 m Kuruvai variety exhibited an RS content of 19%, whereas the Boiled Mapillai Samba variety contained only 8% in its raw state, increasing to 14% after processing (Rajendran and Chandran 2020). These results confirm that heat treatment with moisture enhances RS contents (Van Hung et al. 2020). Given the role of RS in reducing postprandial glucose levels and improving insulin sensitivity, red rice could serve as a dietary intervention for managing metabolic disorders (Amin et al. 2018). However, individual responses to RS consumption can vary, as they are influenced by factors such as gut microbiota composition and overall dietary habits (Fusco et al. 2023).

Red rice demonstrates significant potential as a functional food due to its high bioactive compounds, fiber, and RS levels. However, several challenges remain, including the impact of processing on nutrient retention, variations in bioactive content across studies, and the need for further research on bioavailability. Future studies should focus on optimizing preparation techniques, understanding the mechanisms behind its health benefits, and promoting consumer acceptance to facilitate the integration of red rice into mainstream diets. Addressing these gaps will be crucial in maximizing the nutritional and functional value of red rice and positioning it as a viable alternative to conventional rice varieties.

### 3.4 Pigmented Rice

The profiles of bioactive compounds vary substantially across pigmented rice varieties, explaining the differential efficacy observed in their functional properties. The intensity of the purple–black coloration is directly associated with the concentration of anthocyanins in the pericarp. However, small amounts of carotenoids and chlorophyll also contribute to the bioactive composition of this variety (Choi, Lee 2021; Das et al. 2023).

In contrast, red rice's color is attributed to both the presence of anthocyanins and, more significantly, to proanthocyanidins, polymeric flavonoids with recognized antioxidant activity (Tai and Huang 2021). However, quantitative analyses indicate that proanthocyanidins can reach high concentrations, making them the predominant flavonoid group in red rice. Small amounts of carotenoids, such as lutein, zeaxanthin, β‐carotene, and lycopene, also contribute to the bioactive composition of this variety (Chen et al. 2024).

The primary difference between black and red rice lies in the nature and stability of the predominant phenolic compounds. Anthocyanins, the majority of which are in black rice, have high antioxidant capacity and relative resistance to thermal and oxidative degradation, favoring their preservation during processing and greater bioavailability (Liyanaarachchi et al. 2022). In contrast, the proanthocyanidins in red rice are more prone to polymerization, oxidation, and interactions with proteins and starch, reducing their solubility and absorption, thereby limiting their functional efficacy (Yang et al. 2022; Tyagi et al. 2022). Purple rice, in turn, combines anthocyanins and proanthocyanidins in varying proportions, so its effects depend on the balance between these compounds (Zhu et al. 2022).

Although many studies compare only one pigmented rice variety with WR, few studies simultaneously evaluate all categories of colored rice. This review highlights the limited availability of comparative analyses across varieties, even though some varieties may exhibit more pronounced biological effects than others. Future studies using integrated transcriptomic and metabolomic approaches are needed to identify the molecular pathways and key genes that underpin these differences, providing essential information for selecting and improving pigmented rice cultivars with greater functional potential. To ensure critical comparisons, it is crucial to standardize factors, such as genotype, agronomic conditions, light, temperature, and humidity during experiments.

## 4 Impact of Processing and Cooking Methods on the Nutritional and Functional Properties of Pigmented Rice

The processing and cooking of pigmented rice involve complex physical, chemical, and biochemical transformations that can affect its nutritional composition, bioactive compound retention, and functional properties. Postharvest interventions, such as hulling, milling, parboiling, and drying, determine its final nutrient profile, whereas cooking techniques, including boiling, steaming, roasting, and extrusion, modulate its digestibility, antioxidant activity, and glycemic response.

The review compiled data from studies involving different extraction methods, pigmented rice cultivars, and processing techniques, highlighting the lack of standardization. The functional bioactive compounds present in these varieties can be obtained using conventional and innovative approaches, including radiofrequency‐assisted extraction, supercritical fluid extraction, enzymes, ohmic heating, microwaves, and ultrasound (Kelly et al. 2019).

Each technique has specific advantages and limitations, and the choice of method is influenced by the sensitivity of the compounds, especially anthocyanins, to factors such as temperature, pH, and light (Saloni et al. 2025). Critical factors, such as temperature, extraction time, and solvent type/concentration, determine the efficiency of bioactive recovery, reinforcing the need to identify single or combined methods that maximize yield without compromising functional integrity. Combining techniques can improve efficiency and recovery, whereas extraction followed by microencapsulation helps overcome limitations related to thermal instability, pH instability, and light sensitivity (Saloni et al. 2025).

### 4.1 Processing Techniques and Nutritional Implications

Hulling and milling are critical processing steps that influence the retention of bioactive compounds, dietary fiber, and micronutrients in pigmented rice. Unlike conventional WR, pigmented rice is often consumed in whole or minimally milled form to preserve phenolic compounds, anthocyanins, and flavonoids. However, even minimal milling can cause substantial losses, with reductions of up to 70% in anthocyanin content and significant decreases in essential micronutrients, such as iron and zinc (Sirisoontaralak et al. 2020). Although enzyme‐assisted milling has been proposed to mitigate these losses, its large‐scale application remains limited due to risks of microbial contamination and technical constraints. Genetic and technological advancements, including breeding strategies for improved nutrient retention, offer potential solutions (Kasote et al. 2021).

Parboiling, a hydrothermal treatment, enhances storage stability and nutrient retention by facilitating the migration of phenolic compounds into the endosperm, increasing their bound form and improving stability during cooking. Thermal processing, such as parboiling, promotes gelatinization and retrogradation of starch in the endosperm. In vitro studies indicate that pigmented rice has a longer digestion time than WR due to its lower solubility and swelling capacity, as well as a higher gelatinization temperature (Farooq et al. 2021).

Additionally, parboiled pigmented rice exhibits increased hydrophobicity, which helps preserve essential amino acids, such as tyrosine and phenylalanine (Pal et al. 2018). Despite the degradation of some heat‐sensitive vitamins, parboiling maintains extractable vitamin E. It enhances the bioavailability of bound phenolics, positioning it as a nutritionally favorable alternative to conventional processing methods.

Drying techniques also play a vital role in the nutritional preservation of pigmented rice. High‐temperature drying decreases head rice yield (HRY) and anthocyanin content (Qiu et al. 2021). In contrast, moderate drying temperatures enhance amylose content, water absorption, and starch gelatinization, thereby lowering the GI (Dalbhagat and Misha 2021). Optimizing drying conditions is essential to balance bioactive compound preservation with textural and cooking properties.

Industrial processing alters the phytochemical profile of pigmented rice in complex ways, with effects that vary depending on the nature of the grain and the severity of the treatment. In black rice, more aggressive treatments tend to reduce the levels of heat‐labile compounds (apigenin, catechin, myricetin, and total flavonoids). At the same time, the disruption of cellular structures and the hydrolysis of conjugates can increase the relative detection of more stable flavonoids (kaempferol and luteolin) and some phenolic acids. The sensitivity of anthocyanins to heat treatment is well known. However, specific roasting or extraction conditions, as well as the appearance of degradation products (C_3_G → protocatechuic acid), can mitigate the loss of biological activity. Consequently, the magnitude of changes depends both on the severity of processing (polishing, toasting, microwave, autoclave, etc.) and on factors intrinsic to the grain (genotype, climate, management, and storage), explaining the variability observed between studies (Bagchi et al. 2021; Melini et al. 2019; Aalim et al. 2021).

In red rice, thermal drying and storage have a critical effect on the stability of phenolics and proanthocyanidins. High drying temperatures can initially release matrix‐bound phenolics, but they also favor polymerization, oxidation, and transformations that lead to a net reduction of free and bound phenolics, as well as proanthocyanidins; these losses are exacerbated during storage, especially under warmer and prolonged conditions (Ramos et al. 2022; Ziegler et al. 2018; Lang et al. 2020). However, some studies have observed an increase in certain compounds (caffeic acid) under specific drying conditions (Ferreira et al. 2021), demonstrating that the effects depend strongly on technical parameters and the matrix analyzed.

### 4.2 Effects of Cooking Methods on Nutrient Retention

Cooking significantly affects the phytochemical composition and functional properties of pigmented rice, with black, red, and purple varieties responding differently. In general, thermal processing reduces phenolics and flavonoids, with red rice being more affected because its proanthocyanidins are heat‐labile and prone to polymerization and oxidation. In contrast, the anthocyanins predominant in black rice are more heat‐stable, allowing better preservation of total phenolics and antioxidant activity (Massaretto et al. 2022).

The cooking method and duration further influence the phytochemical profile. In black rice, techniques such as bottom heating, induction, and pressure cooking can reduce flavonoids and anthocyanins while increasing phenolic acids (Zhou et al. 2024). Purple rice shows changes in compounds like citric acid and isocyanates depending on the processing method (Zhu et al. 2022). Thermal degradation is compounded by polymerization, protein and starch complexation, and Maillard reaction products, which reduce solubility and hinder conventional extraction, partially compromising the functional availability of bioactives (Massaretto et al. 2022).

Cooking also impacts starch digestibility and glycemic response. Boiling can cause significant nutrient leaching, whereas steaming preserves water‐soluble phenolics and vitamins (Kodape et al. 2024). Roasting and puffing modify the rice structure, enhancing bioaccessibility of some phytochemicals while maintaining antioxidant capacity. However, excessive heat, especially during boiling, can degrade phenolics by up to 50% due to breakdown of esterified and glycosylated bonds (Bagchi et al. 2021).

Emerging techniques such as vacuum cooking, microwave‐assisted processing, and extrusion offer opportunities to optimize nutrient retention and functional properties. Extrusion, a high‐temperature, high‐pressure process, promotes starch gelatinization and protein denaturation, enhancing the bioavailability of minerals (magnesium, iron, and calcium) while potentially degrading heat‐sensitive vitamins such as thiamine and tocopherols, highlighting the need for process optimization (Kumar and Murali 2020). These approaches are particularly promising for developing gluten‐free, nutrient‐dense functional foods, such as extruded snacks and breakfast cereals.

Anthocyanin retention in purple rice is highly dependent on the cooking method. Autoclaving can cause substantial losses, whereas slow cooking, bain‐marie, or reduced‐immersion cooking better preserves these pigments. Moderate roasting or microwaving can maintain or slightly increase certain compounds, but excessive temperatures or durations result in significant degradation (Nayeem et al. 2021; Fracassetti et al. 2020; Yamuangmorn and Dell 2018; Arora et al. 2021; Yamuangmorn et al. 2021).

### 4.3 Effect of Fermentation on Nutritional and Phytochemical Composition

Brown pigmented rice preserves its bran and germ, making it a naturally more nutrient‐dense grain. Fermentation enhances these attributes by degrading antinutritional factors, such as phytic acid, increasing the bioavailability of minerals and other components (Luo et al. 2023). Furthermore, the process generates bioactive compounds associated with antioxidant, anti‐inflammatory, and probiotic effects. Thanks to this versatility, fermented rice has been incorporated into various food categories, from beverages to snacks, adding flavor and health benefits (Kittibunchakul et al. 2021). Even so, it is crucial to ensure adequate hygiene and sanitary conditions during fermentation to prevent contamination by harmful microorganisms.

Fermentation is one of the most critical food transformation methods, characterized as a biochemical process mediated by microorganisms and their metabolites, such as enzymes and organic acids. This activity promotes the release of bioactive compounds, modifies texture, and alters the structure of cellular components. Among the methods employed, solid‐state fermentation (SSF) stands out for its higher productivity, reduced catabolic repression, and enhanced nutritional and biological properties of foods, resulting in better final quality and lower production costs (Lim et al. 2023).

Microorganisms, such as *Lactobacillus plantarum* and *Saccharomyces cerevisiae*, have been widely used in the fermentation of cereals, vegetables, and fruits, contributing to improved texture and flavor, inhibiting spoilage, and extending the shelf life of foods, such as bread, sausages, and wine (Zhang, Ma et al. 2022). Filamentous fungi, including *Rhizopus oryzae*, *Aspergillus oryzae*, and *Neurospora sitophila*, are known for their high enzymatic capacity and are recognized by the FDA as safe microorganisms for use in food (Yi et al. 2022).

In the case of pigmented brown rice, fermentation with these fungi causes sensory improvements, resulting from the action of metabolites released during SSF and the structural disruption of the grains, favoring greater water absorption, reduced hardness, and the release of volatile compounds responsible for the characteristic aroma and flavor (Mehmood et al. 2023).

Fermentation of BRB with lactic acid bacteria, particularly *L. plantarum*, increased the total phenolic compound content in different milled fractions. Analysis of the phenolic profile revealed a marked increase in protocatechuic, caffeic, syringic, and ferulic acids, as well as catechin. Conversely, a reduction in total anthocyanin content was observed, ranging from 10.80% to 50.38% across fractions, accompanied by a decrease in specific anthocyanins, C_3_G, P_3_G, and cyanidin‐3‐diglucoside (C_3_D) (Lin et al. 2024).

Despite this decrease, antioxidant activity increased in fermented compared to unfermented samples, correlating with the greater availability of phenolic compounds. These findings highlight fermentation as a promising strategy to enrich bioactive components and expand the functional potential of BRB (Lin et al. 2024).

An increase in the total phenolic (from 8.16 to 10.73 mg GAE/g) and flavonoid (from 125.51 to 187.21 mg RE/100 g) content was observed after the fermentation treatments, accompanied by a 28.91% increase in antioxidant activity. Fermentation also improved the nutritional value of black rice flour by reducing antinutritional factors, such as phytic acid (65.65%) and tannins (50.47%), and increasing the concentrations of essential minerals, particularly copper, iron, zinc, and manganese. Although all fermentation treatments enhanced the nutritional and bioactive attributes of black rice, *Lactobacillus brevis* demonstrated a superior effect, improving functional components and attenuating antinutritional compounds (Jan and Kumar 2025).

Fermentation with *L. plantarum* dy‐1 significantly increased the protein and total phenolic compound content of red rice, corresponding to 1.7‐ and 1.4‐fold increases, respectively. An increase in the levels of essential and nonessential amino acids was also observed, accompanied by increased bioaccessibility of phenolic compounds and enhanced antioxidant capacity during in vitro digestion. In addition to these effects, fermentation improved the lipid‐lowering activity of red rice, enhancing its functional potential. These findings indicate that fermentation with *L. plantarum* represents a promising processing strategy for enriching the nutritional and bioactive properties of red rice (Zhu et al. 2024).

Red rice bran varieties had higher contents of phenolic compounds, flavonoids, anthocyanins, and carotenoids compared to WR bran. However, when subjected to SSF with *R. oryzae*, a reduction in these compounds was observed, with decreases of 83.16% and 82.51% in the total flavonoid content for the two varieties tested. Similarly, carotenoid content decreased after fermentation (Janarny and Gunathilak 2020).

Future studies should elucidate the metabolic mechanisms underlying phenolic transformations during fermentation, thereby promoting the broader application of pigmented rice by‐products in the food industry. Additionally, research should focus on standardizing and optimizing fermentation conditions, including the selection of microorganisms, duration, temperature, and humidity, to maximize phenolic enrichment and ensure reproducible industrial processes.

Recurring methodological obstacles include heterogeneity across studies, including different expression units, analytical standards, and extraction/quantification protocols, which prevent direct comparisons and robust meta‐analyses. For the technological applications section to convincingly support the viability of using pigmented rice, future studies must report post‐processing retention data (retention percentages relative to the raw material), exhaustively describe processing parameters (temperature, time, immersion, humidity), and adopt appropriate analytical standards (Li, Wang, et al. 2023). Furthermore, the assessment of bioaccessibility and the presence of degradation metabolites with biological activity should be part of the analytical framework, as such products can contribute to the functional effect of the ingredient after processing.

The effects of processing on anthocyanins, proanthocyanidins, and antioxidant capacity are multifactorial and specific to each variety (black, red, and purple). The systematic inclusion of quantified data on post‐processing bioactivity retention and methodological standardization is a crucial measure for validating industrial applications and guiding the selection of cultivars with greater functional potential.

### 4.4 Safety Considerations and Sustainability

Beyond nutritional implications, processing and cooking practices influence the safety and environmental sustainability of pigmented rice consumption. Rinsing, a common precooking practice, effectively reduces arsenic and mycotoxin levels and depletes water‐soluble vitamins and minerals (Krishnan et al. 2020). The potential for pesticide residues and heavy metal contamination highlights the need for stringent quality control measures, particularly in organically cultivated pigmented rice (Tiozon et al. 2021). During the polishing process, bran is produced, which is often discarded or used as animal feed (Feng et al. 2022). However, bran holds significant potential as a functional ingredient in human food. This potential has expanded alongside the growing demand for healthier and more sustainable products (Gul et al. 2015). Future research and industrial applications should prioritize sustainable processing innovations that enhance nutritional quality while minimizing environmental impact.

Despite advancements in processing and cooking techniques, knowledge gaps remain regarding the interactions between environmental factors, genetic variation, and processing‐induced changes in pigmented rice's nutritional composition. Future research should optimize postharvest and culinary interventions to maximize functional benefits while aligning with consumer preferences for health‐promoting foods. Integrating advanced food processing technologies with targeted breeding programs will further harness the nutritional potential of pigmented rice, reinforcing its role as a valuable dietary staple for human health and well‐being.

## 5 Technological Properties and Food Applications of Pigmented Rice Varieties

### 5.1 Impact of Technology on Changes in Functional Properties

The main results of studies covering technological applications of pigmented rice varieties are summarized in Table 2. The anthocyanins present in purple–red rice bran reduce the digestibility of rice starch by forming V‐type inclusion complexes, established by non‐covalent interactions. This structural modification results in the formation of intra–helical complexes, which directly impact the functional properties of starch products (Zhang, Wang, et al. 2023). Incorporating anthocyanin extracts from purple–red rice bran enhances the antioxidant activity of rice starch. It increases its RS content, thereby reducing the action of digestive enzymes on starch by altering its tertiary and secondary structures (Du et al. 2019). Aromatic amino acids play a central role in the interaction between these enzymes and the extracts, providing the basis for developing starch products with a lower GI (Xu et al. 2021). These findings open up prospects for developing functional foods aimed at glycemic control. Furthermore, exploring encapsulation techniques can help improve the stability and bioavailability of anthocyanin–starch complexes in food formulations (Chet et al. 2020).

Investigating the behavior of other polyphenols, such as flavonoids and tannins, when associated with starch can broaden the understanding of their implications for nutrient digestibility and bioavailability. Exploring the potential prebiotic effect of anthocyanin–starch complexes on intestinal microbiota composition could also be a research focus (Peng et al. 2022). In addition, evaluating the stability of these complexes under various technological processes, including cooking, freezing, and extrusion, is essential to enable their large‐scale application. In vivo investigations are needed to elucidate the physiological impact of these mechanisms on the glycemic response. The composition of anthocyanins in rice bran can vary with factors such as agronomic conditions and processing methods, thereby compromising the reproducibility of results. Finally, integrating anthocyanins into starch products on an industrial scale remains underexplored, requiring further research into stability, economic viability, and consumer acceptance (Zhang, Wang, et al. 2023).

The anthocyanins in red–purple rice bran significantly influence the gluing, rheological, structural, and water migration properties of rice starch. The addition of these anthocyanins reduces the final viscosity of the starch, compromising its ability to gelatinize and form a viscous paste, possibly due to interactions that alter the starch granule structure. In addition, there is an increase in the gel strength and hardness of the starch, as the anthocyanins promote the formation of a firmer gel network within the starch matrix. Non‐covalent interactions between anthocyanins and starch molecules improve particle size distribution, resulting in a more uniform dispersion and potentially affecting the functional properties of starch in industrial applications (Zhang, Zhu, et al. 2023). Although changes in viscosity and gluing properties were observed, which may be beneficial in the creation of gluten‐free or low‐GI foods, it is crucial to investigate the impact of different processing conditions, such as temperature and pH, on the stability of anthocyanin‐red‐purple rice bran–starch complexes during industrial food production (Liu et al. 2021).

The heterogeneous distribution of proteins in the in situ pasting properties of black rice starch influences characteristics such as peak viscosity, setback viscosity, and pasting temperature of starch. These viscosity changes relate to intermolecular interactions between starch molecules and proteins, impacting starch gelatinization, retrogradation, and texture in food products (Chen, Lu, et al. 2022). Protein extraction compromised the structural stability of starch granules, suggesting that proteins play a crucial role in maintaining the physical integrity of starch during processing. Protein–starch interactions contribute to the strength and cohesion of the starch network, thereby affecting its ability to form a gel or maintain texture during processing. Furthermore, the protective effect of proteins on starch granules can prevent excessive starch breakdown during gelatinization, thereby influencing their binding capacity during cooking and processing. The protein distribution in black rice grains occurs mainly in the aleuronic layer, cell membrane, and amyloplasts, with higher concentrations in these regions. This distribution directly influences the properties of rice starch, such as peak viscosity and pasting temperature. Such information is valuable for optimizing food processing techniques, adjusting rice starch for different culinary applications, and controlling the nutritional compositions of grains (Chen, Lu, et al. 2022).

However, some questions remain open despite advances in microencapsulating anthocyanins from purple rice bran using modified glutinous rice starch. Limited exploration of alternative encapsulation matrix materials, such as proteins or biopolymers, could enable a more comprehensive comparison of their efficiency, solubility, and protection (Das et al. 2019). Furthermore, the impact of processing conditions on the bioavailability of anthocyanins after digestion remains to be investigated, as there is a gap in understanding their functionality in real food applications. For a more complete assessment of the long‐term stability of microencapsulated powder, it would be relevant to consider environmental variables such as humidity and exposure to light. Furthermore, the sensory and textural effects of adding microencapsulated powder to final food products are critical factors for consumer acceptance and an essential aspect for future studies on the commercial viability of this technology.

Pigmented rice varieties, such as red and purple rice, have specific technological characteristics that make them relevant for various food applications. Purple rice, for example, has the longest cooking time (31.33 min) due to its amylose content (6.44 g/100 g), which results in greater hardness (173.18 N), making it suitable for products that require a firmer texture. The water absorption rate, which ranges from 2.03 to 2.71, directly impacts the hydration properties and processing of rice. However, the degradation of anthocyanins during cooking can compromise color retention, a crucial factor for the formulation of thermally processed food products. On the other hand, red rice has the highest gelatinization temperature and final viscosity, making it a potential thickener and stabilizer in various food formulations. These differences in grazing and textural properties highlight the multiple applications of pigmented rice in the food industry (Devi and Badwaik 2022). However, variability in water absorption rates may compromise reproducibility, depending on the processing conditions used. In addition, the degradation of anthocyanins, which affects the color intensity of rice during cooking, is a challenge to be overcome to ensure the visual attractiveness of the final products (Tangsrianugul et al. 2019). Despite this, the distinct technological properties of purple and red rice reveal their great potential in food processing, especially in the development of products with modified textures, thickened or stabilized.

Black rice yields increased under optimized ultrasonic immersion conditions (540 W, 45 min, 57°C), with HRY improved by about 20%, reducing rice breakage during hulling. Using ultrasound disrupts the internal structure of rice, promoting hydration and yielding a softer, cooked texture, as well as structural changes that reduce the brittleness of black rice during milling, thereby improving its processing characteristics (Bonto et al. 2020). The method increases the processing efficiency of black rice, making it more suitable for industrial applications. Improved texture and nutritional profile may increase the market value of black rice in functional foods (Wu et al. 2023).

The impact of pretreatment methods (enzymatic hydrolysis, microwave treatment, and immersion soaking with soluble soybean polysaccharides) on textural and cooking properties has been widely investigated. These methods demonstrate a significant improvement in the texture and cooking quality of rice, with enzymatic hydrolysis being the most effective. Enzymatic hydrolysis, for example, significantly reduces the hardness/adhesion ratio, from 107.72 to 26.03, indicating a considerable improvement in grain texture. In addition, it promotes a substantial increase in water absorption during soaking—from 7.76% to 35.60% in 120 min—and reduces cooking time from 30.3 to 23 min, while increasing cooking water absorption from 227% to 284% (Xiong et al. 2023). These treatments are considered promising options for improving rice quality. However, important aspects such as the effects of these methods on long‐term storage and shelf life of rice were not addressed. These variables are crucial for a complete assessment of product quality and its potential for commercial application, as the effectiveness of these methods may vary when applied in real‐world processing environments or across different types of rice (Geng et al. 2020).

The effect of black rice water extract (BRWE) on ground beef burgers has been investigated in several studies, yielding significant technological outcomes. Incorporating BRWE into ground beef burgers has demonstrated significant improvements in meat redness, suggesting that the extract positively influences the product's color, giving it a fresher, more attractive appearance during storage (Prommachart et al. 2020). Furthermore, the addition of BRWE has demonstrated a protective effect against lipid oxidation during storage. Burgers that received BRWE presented significantly lower lipid oxidation levels than the control groups. This effect is crucial for extending the shelf life of ground beef, as lipid oxidation is directly associated with the development of rancidity and loss of sensory quality (Prommachart et al. 2020). However, the concentrations tested to date may be insufficient to assess the effects of higher doses, limiting a full understanding of its efficacy. Furthermore, despite the observed positive results, there is still no detailed understanding of the molecular mechanisms underlying these benefits.

### 5.2 Applications in Food Packaging

Incorporating polyphenol‐rich extracts from purple rice and black rice into chitosan (CS) matrices to develop packaging films with antioxidant and pH‐sensitive properties has been widely studied. Black rice extracts (BREs) contain higher concentrations of total phenols and anthocyanins than purple rice (Yong et al. 2019). However, using concentrations of 3% and 5% of these extracts compromises the structural uniformity of the films, and using 1% extract, whether from black or purple rice, is recommended to ensure the homogeneity of the film matrix (Yong et al. 2019). The formation of hydrogen bonds between CS and the extracts results in substantial improvements in the properties of the films, including water barrier, tensile strength, protection against ultraviolet (UV) radiation, and antioxidant activity (Yong et al. 2019).

The significant presence of anthocyanins gives the films pH sensitivity, enabling color changes in response to pH variations. During pork spoilage monitoring, films with purple rice extracts exhibited more pronounced color changes than those with BREs, demonstrating their potential as active, intelligent packaging in the food industry, with antioxidant properties and the ability to indicate changes in the pH of the packaged product (Shao et al. 2018). However, it is essential to conduct more in‐depth studies on the optimal concentrations of the extracts, as higher concentrations compromise the homogeneity of the film matrix, indicating a limit to the amount of extract that can be incorporated without negatively affecting the film structure (Jiang et al. 2019). Although BRE has a higher total phenol and anthocyanin content, purple rice extract films are more effective for monitoring pork spoilage. This suggests that the efficacy of films as freshness indicators does not depend solely on total anthocyanin content but also on other factors, such as the specific composition of the extract. Therefore, extracts should be selected and prepared to meet specific needs, such as monitoring freshness and offering antioxidant properties.

Technological applications of BRE in indicator films highlight its potential as a natural source of anthocyanins for real‐time monitoring of the quality and freshness of packaged foods. Incorporating BRE into films based on soy protein isolate and CS (SPI/CS) improved antioxidant properties and water barrier capacity, which are essential for increasing the stability of packaged products. However, the excessive presence of anthocyanins compromised the mechanical strength of the film by weakening interactions between macromolecules (Kan et al. 2022). The addition of BRE did not promote significant chemical interactions with the polymer matrix, ensuring the structural stability of the material. Furthermore, BRE's ability to change color in response to environmental conditions reinforces its applicability in developing innovative packaging that provides visual indications of food condition. Although SPI/CS films containing other sources of anthocyanins have demonstrated greater chromatic sensitivity, BRE remains a viable alternative to improve the functional properties of biodegradable indicator films. Further knowledge is needed to optimize the stability and reactivity of BRE‐based films, expanding their applicability in food safety and quality monitoring of packaged foods (Xiao et al. 2023).

Anthocyanins present in BRB (BACNs) have been effectively incorporated into oxidized chitin nanocrystals (O‐ChNCs) and gelatin matrices through non‐covalent bonds, resulting in a uniform distribution of the antioxidant compound within the film structure. Although integrating these anthocyanins into the gelatin matrix decreased the mechanical properties of the films, especially tensile strength, it did not compromise their structural integrity. On the contrary, significant improvements in the microstructure and barrier properties, including resistance to oxygen and moisture, were observed, which are fundamental characteristics for applications in food packaging (Ge et al. 2020). Additionally, these films exhibited remarkable pH sensitivity, with visible color changes when exposed to different buffer solutions, a highly relevant property for monitoring food freshness, as the films can change color with pH shifts during deterioration. This behavior was particularly evident during monitoring of the deterioration of shrimp and skinny tail, where the films changed from purple to shades of blue–gray or brown, indicating product degradation (Ge et al. 2020).

Another notable feature was the greater sensitivity of films with lower BACN concentration to basic volatile amines, substances frequently released during food spoilage. This improved sensitivity makes these films a promising tool for real‐time monitoring of food freshness (Ge et al. 2020). The optimal concentration of BACNs that balances mechanical strength, antioxidant properties, and color change sensitivity remains to be investigated. Future studies should also explore the efficacy of the films in a broader variety of protein‐rich foods and perishable products. Furthermore, the environmental impact of these films’ production, use, and disposal, a crucial issue for their commercial adoption, has not yet been fully evaluated. However, using sustainable materials, such as oxidized chitin and natural anthocyanins from pigmented rice, may represent an environmentally friendly alternative to synthetic plastic packaging, addressing food safety and environmental concerns (Ge et al. 2020).

Incorporating anthocyanin‐rich BRE into biodegradable pectin‐CS films is a promising approach for developing smart packaging. In addition to improving the material's mechanical and structural resistance, these films exhibit a colorimetric response to meat deterioration, changing from red to blue as the degradation progresses, enabling visual monitoring of food quality (Zeng et al. 2022). Despite its potential, challenges still limit its commercial application. Calibration of the relationship between color variation and stage of deterioration requires greater precision to ensure reliability. Limited moisture resistance can compromise film stability in humid environments, and degradation of anthocyanins due to light, temperature, and pH can reduce the indicator's efficacy during storage. Because of this, research continues to advance to improve the stability of bioactive compounds, colorimetric response, and durability of the material, expanding its potential in the sustainable packaging sector and food safety.

The development of smart films incorporating BACNs into a CS/O‐ChNCs matrix has been investigated as a strategy to monitor the spoilage of fresh fish and shrimp. BACNs exhibit color changes from red to grayish‐green over a pH range of 2.0–12.0, evidencing their pH sensitivity. Incorporating BACNs into the CS matrix resulted in good dispersion of O‐ChNCs and BACNs but reduced the mechanical and barrier properties of the films. Despite this reduction, the films demonstrated excellent barrier capacity against UV radiation and antioxidant properties. Films with 3% BACNs were effective for visually monitoring fish and shrimp spoilage, with evident color changes indicating the degradation process (Wu et al. 2019). The decrease in mechanical strength and barrier properties of films represents a significant limitation for their practical applications, especially in contexts that require high durability and barrier effectiveness, such as long‐term food packaging. Furthermore, the behavior and properties of films can vary depending on the rice varieties or pigments used. Using natural materials, such as CS and rice bran anthocyanins, offers an approach to developing more sustainable food packaging, in line with the trend toward the adoption of biodegradable, environmentally friendly materials.

pH‐sensitive CS films combined with oregano essential oil and BRB anthocyanin have been studied as an effective alternative for pork preservation and freshness monitoring. These films showed excellent mechanical strength, UV–Vis light barrier properties, and hydrophobicity. Adding oregano essential oil provided a rapid color change sensitive to pH variations, highlighting its potential as a freshness indicator in food products. When applied to pork, the films improved several quality parameters, such as sensory characteristics, viable microorganism count, pH, TVB‐N value, and color, during storage at 4°C for 12 days. Furthermore, the films reduced the abundance of bacteria associated with spoilage, stress, pathogenicity, and biofilm formation (Hao et al. 2023). Potential variations in results for other food products are unknown, and the long‐term stability and efficacy of the film beyond this period (4°C for 12 days) remain unaddressed. The successful application of the film could be a promising solution to extend the shelf life of perishable products and increase food safety.

Microencapsulation of anthocyanin extract from purple rice bran using modified glutinous rice starch has been widely explored, with attention to its effects on rice dough rheology. Studies demonstrate that microencapsulation of this extract via spray drying yields a powder with favorable physical properties, including solubility (51.83%), particle size (6.44 µm), and low water activity (0.51), thereby ensuring greater stability and thermal resistance. Furthermore, the stability of anthocyanins is significantly improved when stored at 4°C compared to 25°C for 90 days (Das et al. 2019). The increasing addition of microencapsulated powder to rice dough (5%–20%) alters its rheology, reducing pseudoplasticity. These findings highlight the potential of microencapsulation in improving the stability and functionality of anthocyanins in food applications, suggesting new perspectives for developing functional foods enriched with bioactive compounds (Das et al. 2019).

## 6 Functional Effects of Pigmented Rice Varieties: Evidence From In Vitro and In Vivo Animal Studies

Pigmented rice varieties have shown significant functional effects, supported by evidence from both in vitro and in vivo studies (Figure 2). These effects include anti‐inflammatory activity, modulation of the immune system, and potential benefits for osteoarthritis and cancer. Furthermore, pigmented rice has demonstrated promise in enhancing lipid metabolism, protecting against liver injury and hyperuricemia, and reducing oxidative stress. It has also been associated with antidiabetic effects, prevention of diabetic nephropathy, and inhibition of *Helicobacter pylori* infection.

Table 3 summarizes the main results on the effects of pigmented rice varieties from in vitro and in vivo studies. These studies demonstrate functional benefits in cellular and animal models, emphasizing the importance of these varieties for health promotion and disease management.

### 6.1 Anti‐Inflammatory Activity

Black and red rice anthocyanins have demonstrated anti‐inflammatory properties in various in vitro and in vivo models. In an in vitro study using RAW 264.7 cells stimulated by lipopolysaccharides, 100 µg/mL of black rice anthocyanins effectively reduced the production of pro‐inflammatory cytokines TNF‐α and IL‐6 while inhibiting nitric oxide and prostaglandin E2 synthesis, key mediators of inflammation, suggesting a dose‐dependent effect. These effects were associated with suppression of NF‐κB and MAPK signaling pathways, leading to reduced phosphorylation of inflammatory proteins and decreased expression of COX‐2 and iNOS (Qiu et al. 2021). However, the translational relevance of these findings to human physiology remains uncertain, as in vitro conditions may not fully replicate the complexity of in vivo inflammatory responses.

In vivo studies reinforce the potential of anthocyanins in modulating inflammation. In a murine model, a 200 mg/kg dose of anthocyanins from purple–red rice improved intestinal barrier integrity and altered microbiota composition, decreasing intestinal inflammation and toxicity caused by cyclophosphamide (Chen, Shen, et al. 2022). These findings suggest that anthocyanins may protect against chemotherapy‐induced gut damage. However, the mechanisms underlying these effects remain to be elucidated, particularly the direct interaction between anthocyanins and the gut microbiota.

Anthocyanins, key flavonoids responsible for the pigmentation of rice, have been linked to a range of biological activities, including antioxidant (Escher et al. 2020), anti‐inflammatory (Ai et al. 2021), anti‐obesity (Wu et al. 2014), and antitumor properties (Zhou et al. 2018). Despite the promising evidence, one major limitation of current studies is the lack of comprehensive clinical trials assessing their efficacy in human inflammatory conditions. Future research should focus on controlled clinical studies to evaluate bioavailability, metabolism, and long‐term effects.

A mouse ear edema model demonstrated that anthocyanin‐rich extracts from Heishuai black rice modulated the expression of 32 inflammation‐related genes, particularly *IL‐1β*, *IL‐17ra*, *Icam1*, and *Ptgs2*. The downregulation of these genes correlated with a significant reduction in ear edema, further supporting the anti‐inflammatory properties of black rice anthocyanins (Zhao et al. 2021). Although these findings are encouraging, gene expression alterations do not always translate to functional outcomes in human inflammation. Future studies should integrate proteomic and metabolomic approaches to advance understanding of the impacts of anthocyanins on inflammatory pathways.

In autoimmune diseases, such as psoriasis, characterized by chronic inflammation and epidermal hyperplasia, proanthocyanidin extract from red rice showed promising effects. A high concentration (2500 µg) reduced psoriatic symptoms, including redness, thickening, and scaling, by inhibiting the ALOX5 enzyme and reducing the production of inflammatory mediators (Toda et al. 2020). However, such high doses may not be practical for human consumption, directing the need for future evaluation regarding their potential toxicity and molecular mechanisms at elevated concentrations.

Although anthocyanins from black and red rice show compelling anti‐inflammatory potential, the current body of research remains mainly preclinical. Well‐designed clinical trials are crucial to determine their viability as therapeutic agents for inflammatory conditions. Additionally, the synergic interactions between anthocyanins and other dietary bioactive compounds should be explored to assess their cumulative impact on inflammation regulation.

### 6.2 Immune System Regulation

Studies indicate that administering high doses (200 mg/kg) of purple rice bran can modulate the immune response by increasing the production of pro‐ and anti‐inflammatory cytokines, such as IL‐6, TNF‐α, and IL‐10, thereby promoting a balance in immune regulation (Chen, Lu, et al. 2022). Although this effect appears beneficial, it is essential to consider the potential risks associated with immune activation. An increase in the production of inflammatory cytokines can, under certain conditions, lead to exacerbated responses, contributing to chronic inflammation or immune dysregulation. In addition, the increased macrophage activity observed suggests an improvement in immune defense. However, it is still unclear whether this effect is sustained in the long term or could have a negative impact on autoimmune conditions.

Another relevant issue is the extrapolation of these findings to clinical applications. The doses used in experimental models are not always suitable for human consumption, and additional studies are needed to determine safe and effective concentrations in human populations. Compared with other studies on immune modulation by bioactive compounds from purple rice, there remain discrepancies regarding the exact mechanisms involved, underscoring the need for further investigations to validate these effects.

### 6.3 Osteoarthritis Prevention

Osteoarthritis, a degenerative joint disease associated with cartilage degradation, has been the target of studies involving bioactive compounds from purple rice. An extract rich in anthocyanins (100 µg/mL), combined with the metabolites cyanidin and peonidin (10 µM), inhibited the expression of matrix metalloproteinases, enzymes essential for cartilage degradation. This effect was attributed to the suppression of the inflammatory pathways NF‐κB and ERK/MAPK, reducing the IL‐1β‐mediated inflammatory response and the consequent joint damage (Wongwichai et al. 2019).

Although these findings are promising, some critical points should be considered. First, the impact of these concentrations in human models has not yet been adequately studied, and it is necessary to evaluate the compounds’ bioavailability and efficacy at typical dietary doses. Furthermore, although inhibiting the inflammatory pathways NF‐κB and ERK/MAPK is a relevant mechanism in protecting against osteoarthritis, this isolated approach is unlikely to be sufficient for a robust therapeutic effect. Combining these extracts with other therapeutic strategies, such as chondroprotectors or dietary changes, could enhance the reported benefits.

### 6.4 Anticancer Activity

Cancer remains a leading cause of death among noncommunicable diseases, with colorectal cancer ranked as the second leading cause of death and third in global incidence (WCRF 2018). Recent studies have demonstrated the potential therapeutic properties of bioactive compounds, such as anthocyanins, in cancer treatment. For instance, anthocyanins from black rice at a concentration of 0.05 mg/mL increased apoptosis in HCT‐116 cells by 12.1%. Interestingly, when encapsulated in CS and chondroitin sulfate nanoparticles, the apoptosis rate rose to 35.1%. This increase in apoptosis suggests that the bioavailability and efficacy of anthocyanins can be significantly enhanced through nanoparticle encapsulation. Although the results are promising, the in vitro nature of this study limits its applicability to clinical settings. Nanoparticles also raise concerns about the potential toxicity and long‐term safety in human trials. Furthermore, although increases in reactive oxygen species (ROS) and alterations in apoptosis‐related proteins underscore the role of oxidative stress in cancer cell death, these mechanisms are not always specific to cancerous cells, as they can lead to unintended cytotoxic effects on healthy tissues.

Regularly consuming whole grains or foods high in dietary fiber has been associated with reduced risk of developing colon and rectal cancer (Bray et al. 2018). The bioactive compounds in these foods, along with the role of fiber in regulating the intestinal environment, are key factors in their protective effects. However, the protective effect observed in these studies may not be universally applicable. For instance, although fiber benefits many individuals, its impact on health can vary depending on individual gut microbiota composition. Moreover, there is an ongoing debate regarding the optimal amount of fiber for cancer prevention, as excessive intake could lead to digestive discomfort.

Including 10% (21.68 g/100 g) BRB in the diet of cancer‐induced mice led to a significant reduction in colorectal tumor formation, suggesting its anticarcinogenic potential (Budijanto et al. 2023). This study also demonstrated the role of BRB in enhancing antioxidant enzyme activity, which may help mitigate oxidative stress, an established factor in cancer progression. The reported modulation of the intestinal microbiota is particularly noteworthy, as an imbalance in the microbiome is increasingly recognized as a key factor in cancer development. However, despite these promising findings, gastrointestinal side effects in some mice, such as diarrhea and constipation, suggest potential drawbacks of high fiber intake. Additionally, the variation in weight gain among the mice suggests that high‐fiber diets may have differential effects on metabolic health, which could complicate their application in cancer prevention, particularly in individuals with preexisting gastrointestinal conditions.

Although the studies presented promising evidence for the cancer‐protective effects of black rice anthocyanins and fiber, they also highlight the complexities and challenges of translating these findings into practical applications. Future research should focus on addressing the safety, efficacy, and mechanisms of these interventions in human trials and exploring the long‐term effects and potential side effects of high‐fiber diets and nanoparticle treatments.

Evidence suggests that pigmented rice possesses antimetastatic activity, primarily attributed to its phenolic and lipophilic compounds. In the case of black rice, anthocyanin‐rich extracts reduced the expression of metastasis‐related proteins, such as Snail, vimentin, and E‐cadherin (Teng et al. 2020). They inhibited the phosphorylation of FAK, cSrc, and p130Cas, central modulators of cell migration (Pintha et al. 2014). Anthocyanins from this rice have been associated with suppression of the RAF/MEK/ERK pathway and downregulation of MMP‐2 and MMP‐9, metalloproteinases critical in extracellular matrix degradation and metastatic progression (Chen et al. 2015). In red rice, compounds, such as γ‐oryzanol, γ‐tocotrienol, and proanthocyanidins, reduced the secretion of MMP‐2 and MMP‐9, reinforcing their role in inhibiting tumor invasiveness (Pintha et al. 2014).

Taken together, these findings suggest that both black and red rice exert antimetastatic effects by modulating signaling pathways associated with migration, invasion, and extracellular matrix remodeling, thereby strengthening their potential as dietary sources of bioactive compounds for preventing tumor progression.

### 6.5 Lipid Metabolism and Liver Injury Protection

Black rice has been shown to possess multiple beneficial properties, including regulating lipid metabolism and reducing liver damage. Among the various concentrations tested in mice, a 5% BRB supply yielded the most significant results, emphasizing its antioxidant and antidiabetic effects. Specifically, it resulted in notable reductions in glucose levels, enhanced antioxidant activity, reduced weight gain, lowered blood lipid and liver fat levels, and increased insulin sensitivity, all contributing to improved glycemic control. Furthermore, it promoted beneficial changes in the intestinal microbiota by enhancing diversity and stimulating the growth of beneficial bacteria (Li, Du, et al. 2023).

Although these results are promising, a more critical assessment is necessary to understand the broader implications and limitations of these findings. Additionally, although reducing glucose and lipid levels is promising, the long‐term impact of these benefits remains unclear. It is essential to question whether these improvements can be sustained over extended periods of use or are merely short‐term effects. Furthermore, although changes in gut microbiota diversity are beneficial, the specific mechanisms by which BRB exerts this effect remain to be explored. Identifying the key microbial species promoted by BRB and investigating whether these changes are associated with long‐term improvements in metabolic health would be valuable.

### 6.6 Hypouricemic Effect

Hyperuricemia, characterized by elevated uric acid levels in the blood, is linked to various metabolic disorders, including gut and CVD (Vareldzis et al. 2024). The purified fraction of the crude anthocyanin extract from black rice, when administered at a concentration of 0.3 mg/mL, inhibited xanthine oxidase activity in mice. This inhibition was classified as mixed and reversible, suggesting potential for hypouricemic effects and reductions in uric acid levels. Notably, the more concentrated fraction (0.8 mg/mL) exhibited more potent inhibitory effects but raised concerns regarding gastrointestinal discomfort and adverse metabolic interactions, suggesting a dose‐dependent response (Feng et al. 2022). Further studies should identify the threshold beyond which these adverse effects become significant. Additionally, it would be valuable to explore how long‐term administration of black rice anthocyanins at varying concentrations might affect overall metabolic health, including potential risks of chronic side effects.

### 6.7 Antioxidant Activity

A study revealed promising antioxidant effects of black rice anthocyanin extract (BRAE) across various models, including PC12 cells and *Caenorhabditis elegans*. At 50 µg/mL, the extract enhanced the activity of crucial antioxidant enzymes, such as superoxide dismutase and glutathione peroxidase, which are essential for mitigating oxidative stress (Li, Wang, et al. 2023). However, it is necessary to critically evaluate potential confounding factors that may influence the observed results. For instance, although 100 µg/mL exhibited antioxidant properties, it was sometimes accompanied by cytotoxicity. This raises a critical concern about the safety and optimal dosage of anthocyanin extracts in therapeutic applications.

Moreover, in the *C. elegans* model, a 0.5% concentration activated the insulin/insulin‐like growth factor type 1 (IGF‐1) signaling (IIS) pathway, which is associated with longevity and enhanced oxidative stress resistance. However, the 1% concentration did not confer additional benefits and, in some cases, decreased the viability of the organisms (Li, Wang, et al. 2023). This suggests a nonlinear dose‐response relationship, a characteristic that warrants further exploration to understand the mechanisms behind this decline in viability at higher concentrations. It would be beneficial to investigate whether the observed decrease in viability at 1% concentration is due to direct cytotoxicity or a disruption in cellular homeostasis. This could provide insights into the broader implications of using anthocyanin‐based interventions.

Although the antioxidant effects of BRAE show significant potential, the balance between therapeutic efficacy and safety must be carefully considered. Future research should focus on elucidating the mechanisms behind the observed cytotoxicity at higher doses and investigating the pharmacokinetics and long‐term effects of anthocyanin supplementation to ensure its suitability for human use.

### 6.8 Antidiabetic Activity

BRB has been extensively studied for its potential to modulate glucose metabolism and reduce the risk of Type 2 diabetes. The anthocyanins found in black rice, particularly cyanidin‐3‐glucoside, have demonstrated significant antihyperglycemic effects in experimental models. Studies indicate that these compounds can lower blood glucose levels, enhance renal function, and reduce markers of renal fibrosis (Qi et al. 2020). However, although these results are promising, most studies have been conducted in animal models, and there remains a significant gap in human clinical trials. Further research is needed to determine the optimal dosage and long‐term effects in diverse populations.

A key mechanism by which black rice anthocyanins exert their antidiabetic effects is the inhibition of glutamine: fructose‐6‐phosphate amidotransferase (GFAT), which plays a crucial role in glucose metabolism (Bhuyan et al. 2020). This inhibition reduces fructose‐6‐phosphate levels, which may enhance insulin sensitivity and reduce insulin resistance. However, the extent to which this effect translates into meaningful clinical benefits remains unclear, particularly in individuals with early‐stage diabetes. Future studies should focus on the bioavailability and metabolism of anthocyanins in humans, as well as their interactions with other dietary components and medications.

Another notable mechanism associated with the antidiabetic activity of black rice is the inhibition of digestive enzymes, such as amylase and alpha‐glucosidase. Research has shown that BREs inhibit these enzymes more effectively than red rice, leading to improved glucose uptake and reduced hepatic glucose release (Krishnan et al. 2021). However, the impact of black rice consumption on postprandial glucose levels should be evaluated within real‐world dietary patterns, accounting for variations in meal composition, preparation methods, and individual metabolic responses.

The GI of black rice has attracted growing interest due to its potential to mitigate postprandial glucose spikes. Although black rice varieties from the Philippines and Vietnam exhibit low GI values (Pipil et al. 2024), processing, cooking methods, and starch composition influence glycemic responses (Bagchi et al. 2023). The reduction in GI following autoclave and annealing treatments suggests that food processing techniques can be leveraged to optimize the health benefits of black rice (Toontom and Tudpor 2022). However, a deeper understanding of how these modifications affect nutrient bioavailability and overall metabolic health is required. Additionally, GI variability across rice varieties underscores the need for personalized dietary recommendations tailored to individual metabolic profiles.

Comparing black rice to other rice varieties further underscores its potential advantages for glycemic control. Unmilled and pigmented rice varieties generally have lower starch content and higher fiber, protein, and bioactive compound content, contributing to slower carbohydrate digestion and improved glycemic responses (Colasanto et al. 2023). However, although consuming low‐GI foods has been associated with reduced risks of Type 2 diabetes and metabolic syndrome, it is essential to recognize that glycemic responses are influenced by multiple factors beyond GI alone. Combining macronutrient composition, meal timing, and individual insulin sensitivity is crucial for determining overall metabolic outcomes.

The use of pigmented rice flours in food formulations is gaining attention due to their functional properties. Studies have shown that black and red rice flours exhibit varying GI values depending on processing and composition (Waewkum and Singthong 2021). These findings suggest that selecting appropriate rice flours could benefit individuals aiming to manage blood glucose levels through dietary modifications. However, the implications of incorporating pigmented rice flour into commonly consumed food products, such as bread and pasta, require further exploration to assess palatability, consumer acceptance, and long‐term adherence.

Overall, although black rice presents a promising dietary strategy for glycemic control, current research is limited by the lack of large‐scale clinical trials and a comprehensive understanding of its metabolic effects in human populations. Future research should focus on elucidating the synergistic effects of anthocyanins with other dietary components, investigating long‐term health outcomes, and developing evidence‐based nutritional guidelines for individuals at risk of metabolic disorders. Additionally, public health strategies should consider the accessibility and affordability of black rice in different regions to ensure its potential benefits are widely available.

### 6.9 Antimicrobial Activity on *H. pylori*

Black rice may contribute to gastrointestinal health, particularly through its antimicrobial effects, but understanding these effects, especially against pathogens, remains limited. The antimicrobial activity of BRE was evaluated in an animal model, demonstrating efficacy in reducing *H. pylori* infection, especially at 200 mg/kg. Although this dose proved/most effective, some gerbils experienced mild gastrointestinal discomfort, highlighting the importance of balancing the dose with potential adverse effects. The extract at 100 and 200 mg/kg beneficially modulated the infection and improved gastric health, confirming the therapeutic potential of BRE (Kim et al. 2019). However, the observed gastrointestinal discomfort in some gerbils at 200 mg/kg raises concerns regarding the safety profile of this intervention.

Whether these adverse effects result from the bioactive compounds themselves or other factors, such as extract concentration or additional phytochemicals, remains unclear. Furthermore, understanding whether the extract inhibits bacterial adhesion, disrupts membrane integrity, or modulates host immune responses is crucial for determining its practical application.

### 6.10 Hypolipidemic Effect

Hyperlipidemia is a medical condition defined by elevated blood lipid levels, including triglycerides, total cholesterol, and low‐density lipoprotein (LDL) cholesterol. This condition is linked to various metabolic disorders, such as hypertension, insulin resistance, and hepatic steatosis (Ormazabal et al. 2018). Several studies have investigated the potential hypolipidemic effects of pigmented rice varieties, particularly their impact on lipid metabolism and cardiovascular health.

The phenolic extract of Chakhao poireiton rice, at a dose of 400 mg/kg, reduced total cholesterol levels and improved the lipid profile in experimental models by lowering triglyceride levels, raising high‐density lipoprotein (HDL) levels, and decreasing LDL levels. Additionally, it exhibited antihypertensive effects and reduced levels of inflammatory markers, including C‐reactive protein and inflammatory cytokines, in rats. Moreover, it provided hepatoprotective benefits against diet‐induced liver degeneration, reducing lipid infiltration and hepatic inflammation. Regarding cardiac protection, there was a reduction in inflammation and structural damage in cardiac tissue (Chakraborty et al. 2023). However, although these findings are promising, it is crucial to acknowledge that rodent models do not always translate directly to human physiology. The dosages used in animal studies may not be proportionally applicable to humans, and interindividual variability in lipid metabolism must be considered when extrapolating these results.

Brown rice, which retains the bran and germ, is known for its superior nutritional profile and potential role in preventing chronic diseases, such as hypertension, coronary heart disease, diabetes, and metabolic syndrome (Ravichanthiran et al. 2018). However, its lower sensory acceptance has prompted exploration of germination to enhance its nutritional and organoleptic properties. Germination increases the levels of bioactive compounds, such as γ‐aminobutyric acid, ferulic acid, and γ‐oryzanol (Cho and Lim 2016), which are associated with improved metabolic health. The supplementation of a high‐fat diet with germinated brown rice (10%) significantly lowers total cholesterol, triglycerides, and LDL levels in rats compared to non‐germinated brown rice supplementation. Furthermore, germinated brown rice enhances lipase activity and increases ApoA‐I levels, which are linked to improved HDL cholesterol function. Additionally, it reduces inflammatory markers (TNF‐α, IL‐6), modulates the inflammatory response, and positively influences the gut microbiota by increasing beneficial bacterial diversity (Ren et al. 2023). Although these findings suggest a multifaceted mechanism by which germinated brown rice supports lipid metabolism, the literature remains unclear about the long‐term effects in humans and the optimal intake levels required to achieve these benefits.

In another study, black rice supplementation (20%) in a high‐fat diet for hamsters resulted in a significant reduction in total cholesterol (31.6%), LDL (34.6%), triglycerides (36.1%), and hepatic lipids (41.7%). Simultaneously, it increased HDL‐cholesterol (HDL‐c) levels (42.9%) and enhanced antioxidant activity (55%) (Wu et al. 2022). Although these results highlight the potential of black rice as an effective dietary strategy for lipid management, it is crucial to critically evaluate whether these effects are primarily due to its anthocyanin content or other bioactive compounds. Furthermore, the extent to which these benefits are maintained over time and whether they are comparable to pharmacological lipid‐lowering interventions warrants further investigation.

Despite the promising data from in vitro and animal models, there is a pressing need for human clinical trials to confirm the lipid‐lowering effects of pigmented rice. To establish evidence‐based recommendations, bioavailability, metabolic differences among individuals, and dietary interactions must be explored. Moreover, the influence of processing methods, storage conditions, and potential synergistic effects with other dietary components should be considered when assessing the practical application of pigmented rice as a functional food for lipid management. Future research should focus on large‐scale, controlled human studies to provide more definitive conclusions regarding the role of pigmented rice in health and metabolic regulation.

## 7 Health Effects of Pigmented Rice Varieties: Evidence From Human Clinical Studies

Several studies have investigated the effects of consuming pigmented rice varieties on biochemical parameters relevant to human health (Table 4). In addition to these findings, further evidence highlights the broader health benefits of pigmented rice and its associated bioactive compounds, which are summarized in Table 5. This table integrates data from different studies, emphasizing how specific phytochemicals contribute to physiological effects and disease prevention, thereby reinforcing the nutritional and functional relevance of these rice varieties.

**TABLE 3:** Evidence from in vitro and in vivo animal studies of the functional effects of pigmented rice varieties.

Pigmented rice	Objective	Experimental model	Parameters	Main results	Geographical location	References
Purple rice bran	To investigate the effects of anthocyanins from purple rice bran on intestinal barrier function and intestinal microbiota	In vivo	Female mice (SPF)—groups: 50, 100, and 200 mg/kg (low, medium, and high dose)	↑ Intestinal barrier integrity ↓ Inflammatory markers ↑ Beneficial microbiota modulation ↓ Clinical symptoms	Jiangxi, China	Chen, Lu, et al. (2022)
Purple rice bran	To evaluate the immunomodulatory effects of purple rice bran pigments in mice immunosuppressed by cyclophosphamide	In vivo	Female BALB mice—groups: 50, 100, and 200 mg/kg (low, medium, and high dose)	↑ Macrophage activity ↓ Induced immunological damage	Jiangxi, China	Chen, Shen, et al. (2022)
Purple rice bran	To examine the chondroprotective effects of anthocyanin‐rich extracts from Thai purple glutinous rice bran on porcine articular cartilage	In vivo	Anthocyanin extracts: 25, 50, and 100 µg/mL; Cyanidin and peonidin: 1.5 and 10 µM	(−)/⊥ Metalloproteinases ↓ Inflammatory pathways (−)/↓ ROS	Chiang Mai, Thailand	Wongwichai et al. (2019)
Black rice bran	To evaluate the effects of a diet with black rice bran on colorectal cancer in mice induced by dextran sodium sulfate and azoxymethane	In vivo	Diet with 10% black rice bran (21.68 g/100 g)	↓ Colorectal tumors ↑ Antioxidant enzymes ↑ Beneficial microbiota	Bogor, Indonesia	Budijanto et al. (2023)
Black rice bran	To investigate the effect of Zixiangnuo black rice, husked rice, and rice bran on lipid metabolism, liver inflammation, gut microbiota, and metabolite profiles in mice on high‐fat/high‐cholesterol diet	In vivo	Groups: Control, 1%, 5%, 10%, and 15% black rice bran	↓ Weight gain and lipid levels ↑ Hepatic function/↓ Steatosis ↑ Insulin sensitivity ↑ Beneficial microbiota	Jiangsu, China	Li, Du, et al. (2023)
Black rice	To evaluate the hypouricemic effect of black rice anthocyanins in vivo and the possible mechanism of interaction with xanthine oxidase	In vivo	Purified fractions of black rice anthocyanins: 1 (0.0 mg/mL), 2 (0.3 mg/mL), 3 (0.5 mg/mL), 4 (0.8 mg/mL)	(−)/⊥ Xanthine oxidase ↓ Uric acid levels (−)/⊥ Uric acid–producing enzymes	Shaanxi, China	Feng et al. (2022)
Black rice	Identify the molecular mechanisms of the antioxidant activity of black rice anthocyanin extract in PC12 and *Caenorhabditis elegans* cells	In vitro and in vivo	PC12 cells: 10, 50, and 100 µg/mL; *C. elegans*: 0.1%, 0.5%, and 1%	↑ Antioxidant activity (PC12, *C. elegans*, 50 µg/mL/0.5%) ↑ Longevity (*C. elegans*, 0.5%) ↓ Effectiveness at higher doses	Tianjin, China	Li, Wang, et al. (2023)
Black rice	To evaluate the effects of C_3_G on renal dysfunction and fibrosis in streptozotocin‐induced diabetic rats	In vivo	Groups: Normal control (nondiabetic), untreated diabetic group, and diabetic groups treated with C_3_G (10 and 20 mg/kg)	↑ Renal function ↓ Renal fibrosis	Hanzhong, China	Qi et al. (2020)
Black rice	To investigate the anti‐inflammatory effects of black rice anthocyanins obtained by drum drying and extrusion	In vitro	Groups: Control (no treatment), LPS (induced inflammation), and LPS + anthocyanins (25, 50, and 100 µg/mL)	↓ Pro‐inflammatory cytokines and proteins (−)/⊥ Inflammatory markers and signaling pathways	Jiangxi, China	Qiu et al. (2021)
Black rice	To evaluate the inhibitory activity of anthocyanins from black rice bran on the GFAT enzyme for antidiabetic effect	In vitro	Anthocyanin extract: 10, 25, 50, and 100 µg/mL	(−)/⊥ GFAT enzyme ↑ Glucose control/Metabolic effects	Asia	Bhuyan et al. (2020)
Black rice	To investigate the antimicrobial effects of black rice extract on *Helicobacter pylori* infection in *Mongolian gerbils*	In vivo	Black rice extract doses: 50, 100, and 200 mg/kg	↑ Antimicrobial activity (*H. pylori*) ↑ Gastric health ↓ Infection	Wanju, Korea	Kim et al. (2019)
Black rice	Investigating the lipid‐lowering and cardiac risk‐reducing effects of Chakhao poireiton black rice	In vivo	Groups: with and without extract supplementation; concentrations: 200 and 400 mg/kg	↑ Antioxidant activity ↑ Cardioprotective effects ↑ Lipid and histopathological parameters ↓ Inflammatory markers	Manipur, India	Chakraborty et al. (2023)
Black rice	To investigate the anti‐inflammatory action and role in the prevention of chronic diseases of black rice anthocyanins	In vivo	Two varieties: Heishuai (rich in anthocyanins) and AM22 (deficient in anthocyanins); standard diet and diets with 40% whole grain powder of the varieties	↓ Ear edema ↓ Inflammatory genes ↑ Anti‐inflammatory effect	Hanzhong, China	Zhao et al. (2021)
Black rice	To investigate the effect of black rice anthocyanins in chitosan and chondroitin sulfate nanoparticles on apoptosis of HCT‐116 colorectal cancer cells	In vitro	Anthocyanin concentrations: 0, 0.01, 0.02, 0.03, 0.04, and 0.05 mg/mL	↑ Apoptosis (HCT‐116 cells, 0.05 mg/mL + nanoparticles)	Shanghai, China	Liang et al. (2019)
Germinated black rice	To investigate the effects of germinated brown rice on hyperlipidemia and intestinal microbiota	In vivo	Concentration of 10% rice; Groups: normal control, hyperlipidic control, BR (brown rice), GBR (germinated brown rice), BPR (black pigmented rice), and GBPR (germinated black pigmented rice)	↓ Blood lipids ↑ Lipase activity ↑ Beneficial microbiota ↓ Inflammatory markers	Harbin, China	Ren et al. (2023)
Red and black rice	To investigate the role of pigmented rice proanthocyanidins in the regulation of hyperglycemia	In vitro	Two cultivars: black rice (chakhao amubi) and red rice (Njavara); 1 g of black rice and 1 g of red rice in 100 mL of solution	↓ Hyperglycemia ↑ Glycemic control potential	Imphal, India	Krishnan et al. (2021)
Red and black rice	To investigate the antioxidant activity of phytochemicals (phenolics and flavonoids) from different types of rice in protecting HepG2 cells against ABAP‐induced oxidative stress	In vitro	White brown rice, red brown rice, black brown rice, and polished rice; concentrations: 50, 100, and 200 µg/mL	↑ Antioxidant activity ↓ Oxidative stress (HepG2 cells, ABAP‐induced)	Jiangxi, China	Gong et al. (2020)
Black rice	To compare the hypolipidemic effects of pigmented (black) brown rice and nonpigmented brown rice in hamsters on a high‐fat diet	In vivo	Control group: high‐fat diet without supplementation; group supplemented with nonpigmented brown rice; group supplemented with black brown rice; diet: 20% of each type of rice	↓ Total cholesterol, LDL‐C, triglycerides ↓ Hepatic lipid accumulation ↑ Antioxidant mechanisms	Tainan City, Taiwan	Wu et al. (2022)
Red rice	To investigate whether red rice proanthocyanidin inhibits 5‐LOX enzyme activity in vitro and improves inflammatory symptoms in psoriatic skin in mice	In vitro and in vivo	Dose of 50 µL of extract per mouse ear per day; extract concentrations: 25, 250, and 2500 µg; doses: 2, 20, and 200 mg/kg/day	↓ Skin inflammation (−)/⊥ ALOX5 enzyme ↑ Improvement of inflammatory symptoms	Soja, Japan	Toda et al. (2020)

Abbreviations: GFAT, glutamine: fructose‐6‐phosphate amidotransferase; LDL, low‐density lipoprotein; ROS, reactive oxygen species.

**TABLE 4:** Evidence from in vivo human studies of the functional effects of pigmented rice varieties.

Pigmented rice	Objective	Parameters	Main results	Geographical location	References
Artemide and Venere black rice	To assess the impact of pigmented rice on polyphenol and antioxidant levels	Plasma polyphenols, flavonoids, and antiradical power at 30, 60, 120, and 180 min	↑ Plasma polyphenols ↑ Flavonoids ↑ Antiradical power	Vercelli, Italy	Vitalini et al. (2020)
Riceberry rice	Evaluate effects on glycemia, insulinemia, antioxidant status, and appetite	Postprandial glycemia, insulin levels, antioxidant status (FRAP), and appetite ratings	↓ Postprandial glucose (30 and 60 min vs. HMB) ↓ Insulin response (vs. HMB) ↑ FRAP (vs. WB and HMB) → Appetite ratings (no change)	Bangkok, Thailand	Chusak et al. (2020)
Riceberry rice	Investigate the effects of riceberry rice yogurt on postprandial glycemic response, antioxidant capacity, and subjective ratings	Plasma glucose, FRAP, TEAC, ORAC, MDA, appetite ratings	↓ Postprandial glucose ↑ Antioxidant capacity (FRAP, TEAC, and ORAC) ↓ MDA → Appetite ratings	Bangkok, Thailand	Anuyahong et al. (2020)
Purple, red, and brown rice	Evaluate the antioxidant and anti‐inflammatory effects of pigmented rice consumption in humans	Antioxidant activity, malondialdehyde (MDA), TNF‐α, IL‐6	Purple rice: ↑ Antioxidant activity (+70.5%); ↓ TNF‐α (−21.9% at 1 h, −25.4% at 4 h); ↓ IL‐6 (−11.7% at 1 h) Red rice (Yunlu29): ↓ MDA (−9.2% at 30 min); ↓ IL‐6 (−14.1% at 2 h) Brown rice: → no significant effects	New South Wales, Australia	Callcott et al. (2019)
Riceberry rice	To evaluate the effects of riceberry rice beverage (RRB) on postprandial glycemic, insulin, triglyceride responses, inflammatory biomarkers, and antioxidant status	Postprandial plasma glucose, insulin, MDA, triglycerides, FRAP, TEAC, thiol levels, pro‐inflammatory cytokines (IL‐1β, IL‐6, and TNF‐α), appetite sensation	↓ Postprandial glucose, insulin, MDA, triglycerides ↑ Antioxidant capacity (FRAP, TEAC, thiol) ↓ Inflammatory markers (IL‐1β, IL‐6, and TNF‐α) → Appetite sensation	Bangkok, Thailand	Anuyahong et al. (2022)
Riceberry rice	Evaluate the effect of riceberry rice (RR) on postprandial glycaemia	Gastric emptying rate (GER), plasma glucose, insulin, GIP, GLP‐1	↓ GER ↓ Postprandial plasma glucose → Insulin and GLP‐1 ↑ GIP (after WR consumption)	Bangkok Noi, Thailand	Muangchan et al. (2022)
Black rice	Investigate the effects on CVD risk markers in hyperlipidemic individuals	Lipoproteins (HDL, LDL, total cholesterol, and triglycerides), glycemic control (fasting glucose, fructosamine), HDL function (ApoA1, PON1 activities), plasma bile acids	→ Lipoproteins → Glycemic control → HDL function → Plasma bile acids	Norwich, United Kingdom	Aboufarrag et al. (2022)
Fermented glutinous black rice	Evaluate the effect on LDL cholesterol levels	LDL cholesterol levels (mean and median)	↓ LDL cholesterol (experimental group) → LDL cholesterol (control group)	West Java, Indonesia	Fauziyah, Nurjannah, et al. (2020)
Cyanidin‐3‐glucoside‐rich black rice	Assess the effects of C_3_G‐rich black rice extract on cognitive function	Cognitive function (ADAS‐cog, CERAD‐K), subjective memory, safety (hematology, urine)	↑ Subjective memory → Objective cognitive outcomes → No adverse events	Suwon, South Korea	Joo et al. (2019)
Black rice with giant embryo	Assess the effect of black rice on inflammation and metabolic syndrome	Body weight, waist circumference, hs‐CRP (highly sensitive C‐reactive protein)	↓ hs‐CRP → Body weight → Waist circumference	Suwon, South Korea	Joo et al. (2020)
Black rice	Evaluate the effects of BRE on obesity in obese postmenopausal women	Trunk fat, total fat, body fat percentage, weight, BMI, waist circumference, waist‐to‐hip ratio	↓ Trunk fat, total fat, body fat % → Anthropometric measures	Seoul, South Korea	Jung et al. (2021)
Black rice germ and bran supplement	Assess effects of supplementation and exercise on aging biomarkers, physical performance, and muscle strength in older adults	Inflammatory biomarkers (C‐reactive protein, interleukin‐6), endocrine biomarker (IGF‐1), physical performance (walking speed, sit‐to‐stand time), and muscle strength (grip strength)	↓ Inflammatory biomarkers ↑ IGF‐1 ↑ Physical performance ↑ Lower‐body muscle strength → Grip strength	Chiang Mai, Thailand	Seesen et al. (2020)
Fermented glutinous black rice (FGBR)	Assess the efficacy of FGBR in improving lipid profile in dyslipidemia patients	Antioxidant content, fiber content, lipid profile (LDL, HDL, triglycerides, and total cholesterol)	↑ Antioxidants and fiber intake ↑ Lipid profile (improvement in dyslipidemia patients)	West Sumatra, Indonesia	Syarief et al. (2020)
Black rice	Evaluate metabolic effects of black rice anthocyanin extract (BRAE) fortification	Postprandial glycemia, insulin, lipid profile, and lipoproteins	↓ GI of wheat bread (BRAE fortification) → Postprandial glycemia (composite meal) ↑ HDL, Apo‐A1, Apo‐B ↑ Modified LDL/HDL subfractions ↑ Lipid distribution in HDL and LDL	Shaanxi, China	Ou et al. (2023)

Abbreviations: BMI, body mass index; CVD, cardiovascular disease; GIP, glucose‐dependent insulinotropic polypeptide; HDL, high‐density lipoprotein; HMB, Hom Mali bread; LDL, low‐density lipoprotein; PON1, paraoxonase‐1; WB, wheat bread; WR, white rice.

**TABLE 5:** Health benefits of pigmented rice and associated bioactive compounds.

Rice variety	Main bioactive compound	Activity	Health outcomes/Results	Reference
Black rice	Polyphenols and flavonoids	↑ Antioxidant	↑ Plasma polyphenols and flavonoids ↑ Free radical scavenging capacity	Vitalini et al. (2020)
Riceberry bread	Anthocyanins	↑ Antioxidant ↓ Glycemic response	↓ Postprandial glucose (30–60 min vs. HMB) ↓ Insulin response ↑ FRAP → Appetite	Chusak et al. (2020)
Riceberry yogurt	Anthocyanins	↑ Antioxidant ↓ Oxidative stress	↓ Postprandial glucose ↑ Antioxidant capacity ↓ MDA → Appetite	Anuyahong et al. (2020)
Purple rice	Anthocyanins	↑ Antioxidant ↓ Inflammation	↑ Antioxidant activity (+70.5%) ↓ TNF‐α (−21.9% at 1 h, −25.4% at 4 h) ↓ IL‐6 (−11.7% at 1 h)	Callcott et al. (2019)
Red rice	Phenolic compounds	↓ Oxidative stress ↓ Inflammation	↓ MDA (−9.2% at 30 min) ↓ IL‐6 (−14.1% at 2 h)	Callcott et al. (2019)
Riceberry rice drink (RRB)—overweight/obese	Anthocyanins	↑ Antioxidant ↓ Inflammation	↓ Glucose, insulin, MDA, triglycerides ↑ Antioxidant capacity ↓ IL‐1β, IL‐6, and TNF‐α → Appetite	Anuyahong et al. (2022)
Riceberry rice	Fiber and anthocyanins	↓ Glycemic response	↓ Postprandial glucose ↓ GER → Insulin and GLP‐1 ↑ GIP (vs. WR)	Muangchan et al. (2022)
Black rice anthocyanins	Anthocyanins	—	→ Lipoproteins, glycemic control, HDL function, and plasma bile acids	Aboufarrag et al. (2022)
Fermented black rice	Anthocyanins and fiber	↑ Lipid metabolism	↓ LDL cholesterol	Fauziyah, Nurjannah, et al. (2020)
Black rice extracts	Cyanidin‐3‐glucoside	—	↑ Subjective memory → Objective cognitive outcomes	Joo et al. (2019)
Roasted black rice	Anthocyanins and carotenoids	↓ Inflammation	↓ hs‐CRP → Body weight and waist circumference	Joo et al. (2020)
Black rice extract	Anthocyanins	↓ Adiposity	↓ Trunk fat, total fat, body fat % → Anthropometric measures	Jung et al. (2021)
Black rice germ and bran + exercise	Anthocyanins and fiber	↓ Inflammation ↑ Endocrine	↓ CRP and IL‐6 ↑ IGF‐1 ↑ Physical performance and lower‐body strength → Grip strength	Seesen et al. (2020)
Fermented black glutinous rice	Anthocyanins and fiber	↑ Lipid metabolism	↓ Total cholesterol, LDL, and triglycerides ↑ HDL cholesterol	Fauziyah, Aminah, et al. (2020)
Bread fortified with BRAE	Anthocyanins	↑ Lipid metabolism	↓ GI of bread ↑ HDL, Apo‐A1, and Apo‐B ↑ LDL and HDL subfractions	Ou et al. (2023)

Abbreviations: BRAE, black rice anthocyanin extract; GIP, glucose‐dependent insulinotropic polypeptide; HDL, high‐density lipoprotein; HMB, Hom Mali bread; LDL, low‐density lipoprotein; WR, white rice.

### 7.1 Antioxidant Properties and Bioactive Compounds

One study evaluated the effects of acute ingestion of black rice on plasma concentrations of bioactive compounds and antioxidant capacity in healthy individuals, comparing two black rice cultivars with Carnaroli brown rice (control). The results showed that, compared to the control rice, black rice cultivars significantly increased plasma levels of polyphenols and flavonoids after 60 and 120 min of consumption. This increase was accompanied by a notable enhancement in free radical scavenging capacity, suggesting that black rice exhibits more potent antioxidant properties compared to other rice varieties, likely due to its higher content of anthocyanins and other bioactive compounds (Vitalini et al. 2020; Aravind et al. 2021). However, the short evaluation period (up to 180 min of monitoring), the small sample size (19 volunteers), and the analysis restricted to two specific cultivars limit the generalizability of the results. The lack of evaluation of additional health markers, such as markers of inflammation and cardiovascular health, also compromises understanding of the benefits of the rice varieties analyzed.

Similarly, a study investigated the effects of consuming riceberry purple rice bread, rich in anthocyanins, on blood glucose and antioxidant capacity. There was a significant reduction in plasma glucose levels at 30 and 60 min after ingestion compared with Hom Mali bread (HMB), but no significant difference compared with wheat bread (WB). Riceberry rice (RR) bread also resulted in a more moderate insulin response, with lower insulin concentrations at 15 and 60 min after ingestion than with HMB. In addition, riceberry rice beverage (RRB) increased plasma antioxidant capacity when evaluated by the FRAP method, outperforming WB and HMB (Chusak et al. 2020). However, the three types of bread did not significantly affect subjective appetite sensations, such as hunger and satiety. The lack of evaluation of long‐term effects, the lack of measurements of other oxidative stress markers, and the failure to investigate the mechanisms by which riceberry influences glycemia and the insulin response limit the understanding of the real benefits of this rice variety.

A study investigated the consumption of yogurt with RR, showing a significant reduction in plasma glucose levels after 30 min of ingestion, indicating a possible benefit in glycemic control. Plasma antioxidant capacity improved after 60 min of ingestion, with higher incremental areas under the curve (iAUCs) for antioxidant parameters in the riceberry group. In addition, plasma malondialdehyde (MDA), a marker of lipid peroxidation, decreased, suggesting less oxidative damage. In addition, there were no significant changes in subjective feelings of hunger or satiety (Anuyahong et al. 2020). These results indicate that riceberry yogurt can reduce postprandial glucose and improve antioxidant status without affecting appetite. However, the small sample size (19 healthy participants) limits the generalizability of the findings, as the study has only assessed acute effects in the 3‐h postprandial period, without investigating long‐term impacts. The single dose tested (350 g of yogurt) does not allow the evaluation of the effects of different doses or habitual consumption to be extrapolated. Furthermore, the applicability of the results to populations with clinical conditions such as diabetes remains uncertain.

Other randomized clinical studies corroborate the idea that consuming pigmented rice has beneficial effects on the modulation of oxidative stress and inflammation. The antioxidant and anti‐inflammatory effects of acute consumption of pigmented rice were investigated in a randomized clinical trial (RCT), showing significant impacts on oxidative stress and inflammation markers. The consumption of purple rice resulted in a substantial increase in antioxidant activity of 70.5%, an effect maintained throughout all the measurements. In addition, eating this type of rice led to a substantial reduction in levels of the pro‐inflammatory cytokine TNF‐α, with decreases of 21.9% and 25.4% after 1 and 4 h of consumption, respectively. IL‐6 levels were also reduced after consuming purple rice, with a drop of 11.7% after 1 h of consumption (Callcott et al. 2019).

Red rice of the Yunlu29 variety showed a significant reduction in MDA, a biomarker of oxidative stress, by 9.2% (*p* < 0.005) after 30 min of consumption. This effect was complemented by a reduction in IL‐6 levels of 14.1% (*p* < 0.01) at 2 h. These findings suggest that the phenolic compounds present in red rice play a relevant role in modulating oxidative stress and inflammation. On the other hand, the nonpigmented brown rice variety had no significant effect on any of the parameters analyzed (Callcott et al. 2019), suggesting that the observed benefits are directly related to the higher phenolic compound concentrations in the pigmented varieties (Aravind et al. 2021).

The results reinforce the potential of pigmented rice as a functional food capable of modulating antioxidant and inflammatory pathways, thereby reducing the risk of chronic diseases associated with oxidative stress and inflammation. However, the evaluation period of only 4 h makes it impossible to analyze the chronic effects of pigmented rice, and further studies are needed to assess its long‐term impact. The small sample size (*n* = 24) and the exclusion of individuals with metabolic diseases compromise the generalizability of the findings. In addition, the bioavailability of polyphenols was not investigated, leaving the role of their metabolism in the observed effects uncertain.

### 7.2 Glycemic Control and Postprandial Metabolism

Studies on the impact of fermented RR in overweight and obese populations also indicate benefits in glycemic control and the modulation of metabolic parameters. The study on RRB demonstrates its positive effects on postprandial metabolic responses in overweight and obese men. When consumed with a meal high in carbohydrates and moderate in fat, RRB significantly reduced the incremental area under the curve for plasma concentrations of glucose, insulin, MDA, and triglycerides. In addition, RRB increased antioxidant capacity and reduced inflammation, with lower levels of pro‐inflammatory cytokines (IL‐1β, IL‐6, and TNF‐α) observed after RRB consumption. However, no significant difference in appetite‐related sensations was observed between meals with and without RRB (Anuyahong et al. 2022). RRB can improve postprandial glycemic control, lipid metabolism, and inflammation in overweight and obese individuals while simultaneously improving antioxidant status. The findings were observed in a small sample, without long‐term follow‐up, and did not indicate effects on appetite regulation.

The acute effects of RR consumption indicated that RR intake delayed the gastric emptying rate compared to WR, significantly reducing postprandial plasma glucose concentrations. This suggests that RR, due to its higher fiber content, is more effective at moderating postprandial glycemia by delaying gastric emptying. Despite the reduction in plasma glucose, RR and WR did not differ significantly in plasma insulin or GLP‐1 concentrations. However, a notable difference was observed in glucose‐dependent insulinotropic polypeptide (GIP) plasma concentrations. After WR ingestion, plasma levels of GIP increased markedly compared to those after RR consumption. This suggests that the slower gastric emptying associated with RR may influence GIP secretion and subsequent carbohydrate absorption, contributing to glycemic control (Muangchan et al. 2022). However, the small sample size (*n* = 6), the short‐term nature of the study, and the single‐dose experimental design limit the extrapolation of the results to the general population. In addition, the effects of regular riceberry consumption on glycemic control were not evaluated. Investigating the role of its bioactive compounds in modulating glycemia, especially its antioxidant properties, could provide a broader understanding of its metabolic impacts.

Evidence indicates that phenolic‐rich extracts of black rice delay starch digestion by inhibiting carbohydrate hydrolases (pancreatic α‐amylase and α‐glucosidase) in the small intestine (Thilavech et al. 2025). Among phytochemicals, C_3_G stands out for inhibiting α‐amylase more strongly than P_3_G. Docking studies indicate that C_3_G forms hydrogen bonds in the active site of α‐amylase, with Glu233 playing a critical role, thereby reducing catalytic activity (Thilavech et al. 2025).

Regarding α‐glucosidase, C_3_G selectively inhibits sucrase (with no effect on maltase), anchoring to specific residues (Leu313, Ser157, Tyr158, Phe314, Arg315, and Asp307) via seven hydrogen bonds and four hydrophobic interactions; Tyr158 serves as the primary anchoring point. These interactions induce conformational changes that explain the decrease in enzyme activity (Xue et al. 2021). Together, these mechanisms delay glucose release into the intestinal lumen and attenuate the postprandial glycemic peak, offering a plausible molecular basis for the metabolic effects attributed to black rice and its anthocyanins.

BRE itself did not inhibit glucose uptake in the Caco‐2 Cell model; however, C_3_G exhibited an inhibitory effect on glucose uptake in this model (Barik et al. 2020). The lack of inhibition by BRE may be due to its lower C_3_G content. Molecular docking simulations suggest that C_3_G interacts with two major hexose transporters in enterocytes: sodium‐dependent glucose transporter 1 (SGLT1) and glucose transporter 2 (GLUT2), which are crucial for glucose absorption across the intestinal barrier (Barik et al. 2020).

In another study, increased glutathione peroxidase activity was observed in obese women who consumed black rice flour substitutes for 6 weeks, with no impact on SOD. Similarly, 12‐ to 24‐week interventions confirmed increased total antioxidant activity compared to controls, suggesting a potential mechanism for attenuating oxidative stress and preserving endothelial function (Kim et al. 2008).

Regarding glucose metabolism, a slight but statistically significant reduction in fasting glucose was observed in obese postmenopausal women after supplementation with BRE (Jung et al. 2021). However, these effects are of limited clinical relevance in normoglycemic populations, suggesting that more significant benefits may occur in individuals with hyperglycemia (Mendoza‐Sarmiento et al. 2023). Regarding lipid profile, the most consistent evidence points to reductions in total and LDL cholesterol when pigmented rice is consumed as a whole food. At the same time, the effects on triglycerides, body weight, and body mass index (BMI) remain nonsignificant (Ou et al. 2023).

### 7.3 Cardiovascular and Lipid Profile Effects

A study investigating the effects of anthocyanins derived from black rice on CVD risk factors in hyperlipidemic individuals reported no significant changes in the main measured biomarkers. Participants consumed 320 mg of anthocyanins from black rice daily for 28 days in a randomized, placebo‐controlled, double‐blind, and crossover trial. The results showed that, compared to placebo, there were no significant changes in lipoprotein levels (total cholesterol, HDL, LDL, and triglycerides), glycemic control (such as fasting glucose and fructosamine), and biomarkers associated with HDL function, including ApoA1, HDL3, and paraoxonase‐1 (PON1) activities, such as arylesterase and lactonase. In addition, no changes were observed in plasma bile acids, which are also considered relevant metabolic health indicators. These findings suggest that the intake of anthocyanins derived from black rice, at the dose tested, did not lead to improvements in lipid profile and glycemic control in the short term (Aboufarrag et al. 2022), challenging the assumption that anthocyanins can have a substantial impact on CVD risk factors in the short term.

The intervention with fermented black rice led to a significant reduction in LDL‐cholesterol levels. The group consuming 200 g of black glutinous rice daily, combined with low‐fat diet counseling, showed a notable decrease in their average LDL cholesterol levels. Specifically, the average LDL cholesterol in this group dropped from 157.47 mg/dL before treatment to 142.32 mg/dL after treatment (Fauziyah, Nurjannah, et al. 2020). More longitudinal studies would be needed to assess the sustained impact of fermented black rice on LDL cholesterol levels over a more extended period. The biochemical processes underlying this effect remain unclear, and other potential confounding factors, such as health conditions, physical activity levels, and other dietary habits, may independently influence cholesterol levels.

Consuming snack bars made from fermented black glutinous rice (FGBR) significantly improves lipid profiles in individuals with dyslipidemia. Significant differences in total cholesterol levels, triglycerides, LDL cholesterol reduction, and HDL cholesterol were observed, demonstrating the efficacy of modulating blood lipids. The decrease in LDL cholesterol and triglyceride levels can be attributed to the high content of antioxidants and fiber present in FGBR. Consuming foods rich in anthocyanins is associated with improved lipid metabolism, reduced obesity risk, and better glycemic control (Fauziyah, Aminah, et al. 2020). These bioactive compounds have been shown to positively influence adipokine gene expression, modulating inflammatory processes and promoting metabolic homeostasis. The positive effects observed on HDL‐c corroborate previous findings on the impacts of flavonoids on endothelial function. The anthocyanins in black rice have antioxidant properties that promote endothelial vasodilation, reducing inflammation and increasing nitric oxide bioavailability, an essential factor for vascular health (Trinovani et al. 2020). In addition, the intake of soluble fibers in FGBR contributes to reducing cholesterol absorption in the intestine and to improving bile excretion, favoring a healthier lipid balance.

The in vivo effects of bread fortified with BRAE were explored through two RCTs involving 24 healthy participants each (Ou et al. 2023). The 2‐h postprandial test with BRAE significantly reduced the GI. However, this reduction in GI did not translate into a statistically significant improvement in postprandial blood glucose levels, indicating that the effects of BRAE on glycemia may be influenced by individual variability. Specifically, fortification with BRAE improved plasma levels of HDL‐c, Apo‐A1, and Apo‐B, with statistical significance for all three markers. In addition, changes in LDL and HDL subfractions, as well as remodeled lipid distributions within LDL and HDL particles, were observed, supporting the potential of BRAE to modulate lipid metabolism. Although the effects of BRAE on postprandial glycemia were modest and varied among individuals, anthocyanin fortification showed promising effects on lipid profile, particularly in improving HDL‐c, Apo‐A1, and Apo‐B concentrations. The small sample size may not have been sufficient to detect subtle metabolic impacts. Furthermore, the heterogeneity in the postprandial polyphenolic profile suggests that individual variations in the bioavailability of these compounds may have influenced the results.

Evidence from intervention studies suggests that consuming pigmented rice may have beneficial effects on antioxidant activity and lipid profile, although clinical results remain inconsistent and of limited magnitude. In patients with coronary artery disease, Wang et al. (2007) reported a significant increase in antioxidant capacity, as assessed by FRAP, after 6 months of intervention with black rice fractions, with no change in superoxide dismutase activity.

### 7.4 Anti‐Inflammatory and Cognitive Effects

BREs rich in cyanidin‐3‐glucoside (C_3_G) significantly impacted subjective memory in older adults with subjective memory impairment after 12 weeks of supplementation (Joo et al. 2019). Although subjective memory improved remarkably, the study found no statistically significant differences in objective cognitive outcomes. The effects of the extract on more formal cognitive tests may not be as pronounced as those on perceived memory. In any case, it has potential as a dietary supplement for individuals concerned about memory decline.

The effects of roasted black rice with a giant embryo (BR) compared to WR on various metabolic parameters in adults with metabolic syndrome were investigated. Although consumption of BR and WR showed no significant differences in body weight changes or reductions in waist circumference, a notable result was a decrease in serum highly sensitive C‐reactive protein (hs‐CRP). The BR group showed a significant reduction in hs‐CRP levels (−0.110 mg/dL), whereas WR showed a slight increase (0.017 mg/dL), indicating that BR had a more potent anti‐inflammatory effect than WR (Joo et al. 2020). The phytochemicals in BR, such as anthocyanins and carotenoids, may play a critical role in alleviating systemic inflammation, regardless of changes in body weight. The chronic low‐grade inflammation contributes to CVD and other chronic conditions (González‐Chávez et al. 2018). However, the study used a single dose of roasted black rice powder diluted in water, without considering variations in preparation methods or the effects of different forms of consumption (whole grains, cooked rice) on metabolic parameters. Including inflammatory biomarkers, such as interleukins and tumor necrosis factors, could provide a more detailed view of black rice's anti‐inflammatory effects and its potential to manage metabolic syndrome.

Supplementation with BRE has also shown positive effects on the body composition of obese postmenopausal women. A study reported a reduction in adiposity in this target group over 12 weeks, with significant decreases in trunk fat, total fat, and body fat percentage in the BRE group compared with the placebo group (Jung et al. 2021). These findings were obtained through dual‐energy x‐ray absorptiometry (DXA) evaluations, which showed that daily consumption of 1 g of BRE could favorably influence participants’ body composition. However, anthropometric parameters assessed by bioimpedance, such as body weight, BMI, waist circumference, and waist‐to‐hip ratio, showed no significant differences between the groups. This suggests that the effects of BRE supplementation were more specific to reducing body fat than to altering total body mass. The short duration of the intervention (12 weeks) prevented assessment of the long‐term effects of BRE supplementation on body composition. Furthermore, the absence of metabolic biomarkers restricted a more in‐depth analysis of its impact on obesity.

The combined intervention of the black rice germ and bran supplement with a physical exercise program significantly modulated inflammatory and endocrine biomarkers. It improved physical performance and muscle strength in elderly participants. There was a significant reduction in C‐reactive protein and interleukin‐6 levels, indicating reduced systemic inflammation, and a substantial increase in IGF‐1, a hormone crucial for maintaining muscle mass and tissue recovery (Seesen et al. 2020). Regarding physical performance and muscle strength, the combined intervention led to significant improvements in transitioning from sitting to standing and in gait speed. Although a trend toward improvement in handgrip strength was identified, this parameter did not show a statistically significant change over the 24 weeks of intervention (Seesen et al. 2020).

The findings reinforce the hypothesis that combining the consumption of black rice germ and bran with a physical exercise program can have synergistic effects in promoting healthy aging. The improvements observed in lower limb muscle strength and in inflammatory and endocrine biomarkers suggest that this approach can help maintain mobility and reduce chronic inflammatory processes in the elderly (Seesen et al. 2020). However, the lack of significant improvement in handgrip strength may be associated with the low intensity of the training. At the same time, the 24‐week duration may have been insufficient to induce significant changes.

### 7.5 Limitations

Despite these promising findings, most studies are short, have small sample sizes, and are highly heterogeneous in their methods, limiting the generalizability of their results. Therefore, little is known about the effects of prolonged consumption of pigmented rice and, above all, about its long‐term safety. This is particularly relevant because, unlike polished rice, pigmented rice is generally consumed whole, preserving bioactive compounds but also potentially toxic elements.

The concentration of heavy metals, such as arsenic, cadmium, and lead, in grains is directly related to soil characteristics, particularly pH and organic matter content, which are determining factors in plant mineral absorption (Liu et al. 2024). Furthermore, age, sex, and milling degree modulate dietary exposure, thereby varying the risk of chronic ingestion across populations (Peng et al. 2020; Jiang et al. 2022).

In this sense, the viability of incorporating pigmented rice as a functional food depends not only on proving its metabolic benefits but also on defining safe and effective long‐term consumption levels, implementing monitoring systems for the presence of metals, and standardizing the content of anthocyanins and other bioactive compounds. Advances in this field require larger scale and longer term RCTs conducted in populations at higher metabolic risk, as well as safety and tolerability studies that consider the agricultural origin and grain processing. Although current data support the potential of pigmented rice as a functional food, the lack of robust evidence on long‐term consumption, cumulative toxicity, and practical applicability limits definitive conclusions, reinforcing the need for integrated approaches that address efficacy, safety, and feasibility for public health use.

## 8 Conclusions and Future Perspectives

Pigmented rice varieties, including black, red, and purple rice, have attracted increasing attention due to their high nutritional value and diverse bioactive compounds, positioning them as promising options for the agro‐food sector. These varieties and their by‐products are rich in phenolic compounds, dietary fiber, and RS, which contribute not only to their nutritional profile but also to their functional properties. However, the composition and concentration of these bioactive compounds can vary significantly among different cultivars, underscoring the need for more systematic characterization to enable reliable comparisons and guide their application in food products.

Scientific evidence from in vitro, preclinical, and clinical studies has highlighted the health‐promoting potential of pigmented rice, particularly in preventing chronic diseases. Despite these advances, significant knowledge gaps remain. Future research should optimize processing and storage conditions to preserve bioactive compounds, such as anthocyanins, and to understand their bioaccessibility and bioavailability in humans better. Furthermore, the molecular mechanisms by which these bioactives exert physiological effects, including modulation of glycemic control, antioxidant activity, and metabolic pathways, warrant further investigation.

In addition to their health benefits, bioactive compounds in pigmented rice have been shown to improve technological properties, including stability, viscosity, and texture, and to support the development of biodegradable and smart packaging films. However, research on the long‐term stability, sensory acceptance, and industrial scalability of these applications remains limited. Understanding these factors is essential for effectively integrating pigmented rice into functional food formulations and for developing novel, value‐added products that meet consumer expectations.

Addressing these gaps will not only strengthen the evidence supporting the functional and technological potential of pigmented rice but also promote its broader adoption as a sustainable, health‐promoting ingredient across domestic and industrial contexts. By focusing on these research priorities, future studies could provide the foundation for evidence‐based recommendations, enhance the commercial and nutritional value of pigmented rice, and expand its role in innovative and sustainable food systems.

## Author Contributions

**Adolfo Pinheiro de Oliveira**: conceptualization, investigation, writing – original draft, methodology, validation, visualization, data curation, formal analysis. **Thatyane Mariano Rodrigues de Albuquerque**: conceptualization, investigation, writing – original draft, methodology, validation, visualization, writing – review and editing, formal analysis, supervision. **Evandro Leite de Souza**: conceptualization, investigation, writing – review and editing, project administration, supervision, data curation, resources.

## Conflicts of Interest

The authors declare no conflicts of interest.

## Data Availability

The data were used for the research described in the article.
